# Consistent root microbiomes across contrasting habitats in a wild perennial vine

**DOI:** 10.1111/plb.70245

**Published:** 2026-07-06

**Authors:** K. Aleklett, K. Karlsson Green, C. B. Andersen, N. Ramirez, D. Kadish, L. Grenville‐Briggs, Å. Lankinen

**Affiliations:** ^1^ Department of Plant Protection Biology Swedish University of Agricultural Sciences Lomma Sweden; ^2^ Department of Biology Lund University Lund Sweden; ^3^ School of Arts and Communication Malmö University Malmö Sweden

**Keywords:** AMF, multitrophic interactions, phenotypic plasticity, plant microbiome, plant performance, rhizosphere, soil nutrients, *Solanum dulcamara*, urban microbiome

## Abstract

- While we are beginning to understand that the plant microbiome is important for plant health, we still lack information about how plant microbiomes are shaped across environments and how they influence plant performance, in particular, in wild study species.
- Here, we examined the root microbiota of the perennial vine *Solanum dulcamara*, a wild relative of potato with an unusually wide ecological amplitude. Using amplicon sequencing, we characterized root communities of bacteria, fungi, and arbuscular mycorrhizal fungi in eight populations across four habitat types (beach, forest, rural, urban) and investigated the link with habitat and plant performance.
- We found significant differences in the composition and diversity of the root microbiota across habitats and populations, but a core set of taxa (61% of all bacterial and 73% of all fungal) made up the majority of the root microbiome. The microbiome composition was connected to soil pH and plant nutrients. Even though the investigated populations differed in herbivory and plant performance, the association between plant performance and microbial composition was weak.
- In conclusion, our results suggest that wild species can have similar root microbiomes across widely different habitats, and that plant performance is not always directly linked to the plant microbiome.

## Graphical abstract

Communities of bacteria and fungi associated with the roots are similar across diverse habitats in a wild plant.

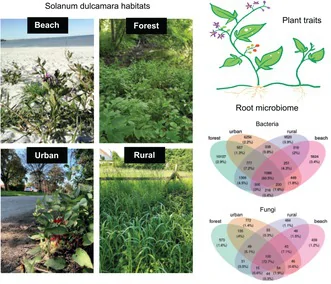

## INTRODUCTION

Plants are considered holobionts and home to a diverse variety of beneficial, commensal, and pathogenic microorganisms (Hassani *et al*. 2018). The plant microbiome is formed by microorganisms in or near a plant, in particular inside or outside the roots or leaves (Chialva *et al*. 2022). Plant‐associated microbes benefit from protected, nutrient‐rich habitats, while host benefits include positive effects on plant health, the acquisition of nutrients, stress tolerance, and fitness (Cordovez *et al*. 2019; Trivedi *et al*. 2020; Singh *et al*. 2025). A better understanding of how microbiomes are shaped could therefore support efforts to control and/or manipulate plant microbiomes in plant production systems to increase yield and improve plant health (Santos & Olivares 2021; Chialva *et al*. 2022).

One of the long‐standing questions in plant microbiome research has been to what extent it is the plant, or the surrounding environment in which the plant is growing, that determines the composition of its microbiome (Dastogeer *et al*. 2020). While multiple studies have seen both species and genotype differences in the root microbiota (Bulgarelli *et al*. 2012; Aleklett *et al*. 2015; Wagner *et al*. 2016; Brown *et al*. 2020; Lazcano *et al*. 2021), results from several studies also suggest that the local environment where the plant grows, for example, soil conditions, can determine the composition of the root microbiota (Wagner *et al*. 2016; Thiergart *et al*. 2020; Vieira *et al*. 2020). In addition, a study on microbial communities in root systems of *Arabidopsis thaliana* and related plant species in the Brassicaceae family (Vieira *et al*. 2020) suggested that the root microbiota might be divided into two parts, a ‘conserved core’ and an ‘environment‐responsive sub‐community’, as plant species only explained a small fraction of the variation among microbiomes.

Microbes, such as nitrogen‐fixing rhizobia and arbuscular mycorrhizal fungi (AMF), have the ability to affect functional plant traits (reviewed by Friesen *et al*. 2011). AMF extends beyond the plant microbiome, into the soil and forms mutualistic symbioses supporting the nutrient uptake of the plant, mainly trading phosphorus in exchange for organic carbon (Smith & Read 2008), as well as providing increased stress tolerance (reviewed by Begum *et al*. 2019) and water uptake (Kakouridis *et al*. 2022). While less is known about whether whole microbiomes are linked with phenotypic trait variation in plants across habitats, and how they impact host evolution (Cordovez *et al*. 2019; Fitzpatrick *et al*. 2020; Kolodny & Schulenburg 2020), differences in the rhizosphere microbiome have been connected to host resistance *versus* susceptibility to selected soil pathogens (Lazcano *et al*. 2021). Furthermore, Rolli *et al*. (2014) detected that the root microbiome of grapevine was able to mediate drought stress by inducing phenotypic changes in their host plants, such as increasing shoot length and photosynthetic activity, under dry conditions. Thus, plant phenotypic traits may be a useful predictor of microbiome function, implying that a focus on comparing microbiome variation with more detailed measures of the plant hosts could help us better understand microbial inheritance in the plant microbiome (Wagner 2021).

Plant microbiome research has so far mainly focused on examining microbiomes in model species, such as annual *Arabidopsis thaliana* (Lundberg *et al*. 2012; Bulgarelli *et al*. 2015; Tkacz *et al*. 2015), or commercially important crops in agricultural systems (*e.g*., tomato: (Ottesen *et al*. 2013); corn (Pfeiffer *et al*. 2017); rice: (Edwards *et al*. 2015); potato: (Andersen *et al*. 2024)). Hence, to add to the larger picture of how plant microbiomes are shaped and operate, studies across diverse sets of species and habitats in more complex natural ecosystems need more attention (Berg *et al*. 2017; Pérez‐Jaramillo *et al*. 2018). For example, studies on the core set of microbiota across diverse natural environments are relatively rare, showing a range of 5–45% (Lasa *et al*. 2019; Thiergart *et al*. 2020; Ortúzar *et al*. 2024; Cui *et al*. 2025). Moreover, there is an increasing awareness of the impact that anthropogenic activities and alterations to natural ecosystems have on microbial communities, and a growing need to take this into consideration in plant microbiome research and management (Berg & Cernava 2022). One such example are urban environments that are known to have more impervious surfaces, higher summer temperatures, less vegetation (Santangelo *et al*. 2022), with soils that are more compressed, polluted and with higher pH and salinity (Asabere *et al*. 2018; Hanfi *et al*. 2020; Dmuchowski *et al*. 2021) than rural environments. In comparisons across environments, it would thus be of great interest to include studies of the composition and diversity of wild plant microbiomes in urban environments, which, to the best of our knowledge, have not been thoroughly investigated.

*Solanum dulcamara* L. (Bittersweet nightshade) is a perennial woody vine native to Eurasia, and a wild relative of commercially important crops such as potato and tomato. An interesting feature of *S. dulcamara* is that it grows and thrives in extremely variable environments – from beaches to roadside ditches and swampy understories in the forest (Golas *et al*. 2010). It also spreads and proliferates into urban spaces, for example, in shrubberies. *S. dulcamara*, therefore, provides a unique opportunity to examine how the root microbiome of a single species is related to the local environment across a wide spectrum of habitats, as well as to plant performance traits (*i.e*., measurable traits that indicate plant growth and reproduction).

While the existence of *S. dulcamara* sun and shade ecotypes has been suggested, with different abilities to use light efficiently for photosynthesis (Gauhl 1979), other studies rather indicate low genetic diversity between populations (*e.g*., Golas *et al*. 2010; Poczai *et al*. 2011) and instead, high levels of phenotypic plasticity in response to habitat differences (Zhang *et al*. 2016), for example, regarding production of adventitious roots (Dawood *et al*. 2014; Zhang *et al*. 2017), or growth patterns (Savinykh & Konovalova 2020). With regards to ecological interactions, *S. dulcamara* is known to host a diverse invertebrate community that includes several known antagonistic species (herbivores) and beneficial species (natural enemies and pollinators) (Symon 1979; Calf & van Dam 2012), which thus could have either positive or negative effects on plant performance. There is also the possibility of indirect interactions between species in the *S. dulcamara* food web that could affect plant performance. For example, AMF have been suggested to influence the regulation of chemical defences against insect herbivores in *S. dulcamara* (Minton *et al*. 2016). It is therefore of interest to investigate how the degree of herbivory and the composition of AMF communities vary across the diverse habitats that *S. dulcamara* inhabits, and whether it is linked with plant performance and the composition of the rest of the plant microbiome. It could further be of commercial interest to better understand microbial community dynamics in the root microbiome of *S. dulcamara*, as it has been shown that the *S. dulcamara* rhizosphere can host and provide an overwintering habitat for *Phytophthora infestans* – the economically important pathogen known to cause potato late blight (Vetukuri *et al*. 2020). For example, there is an increasing interest in rewilding through harnessing the microbiomes of wild ancestral plants to boost those of our agricultural crops (Pérez‐Jaramillo *et al*. 2016; Chen *et al*. 2021; Raaijmakers & Kiers 2022).

**Fig. 1:**
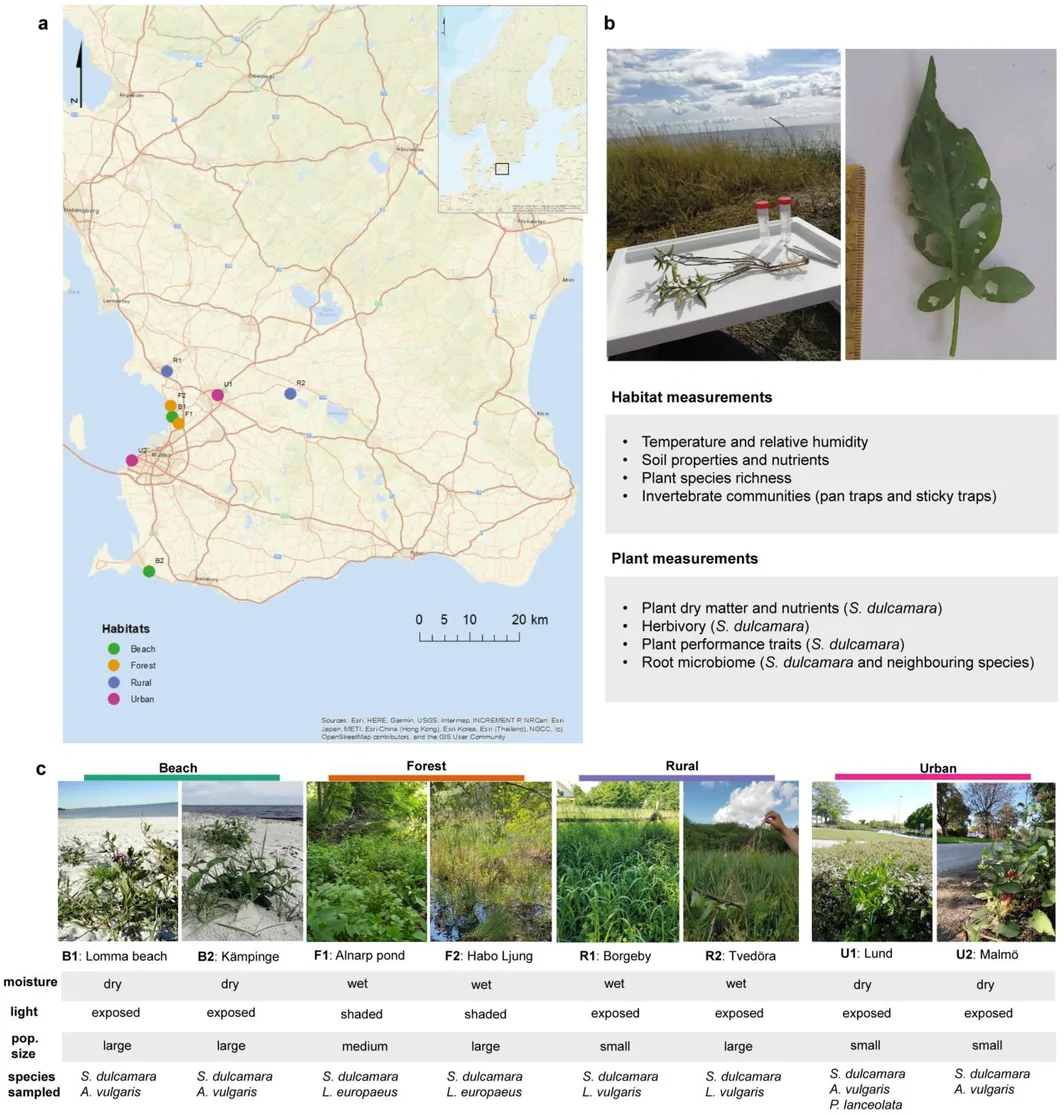
Overview of the eight populations of *Solanum dulcamara* included in this study and performed measurements. (a) Map of Scania, southern Sweden, showing population locations and habitat type (for exact coordinates, see Table S1). Map of Sweden in the top right corner. (b) Habitat and plant measures conducted in the study. Top figures show field set‐up for root microbiome sampling (left) and herbivory of *S. dulcamara* leaf (right). (c) Table showing pictures from the different *S. dulcamara* populations and listing moisture and light conditions for each population used for habitat classification, as well as population size and which additional plant species were selected for root microbiome sampling.

To study the composition and diversity of the root microbiome across the wide ecological amplitude of *S. dulcamara* and its possible link to plant performance traits, we surveyed eight wild populations in southern Sweden across four different habitat types (classified as *beach*, *forest*, *rural, and urban*) (Fig. 1). We compared the microbial root communities (bacteria, fungi, and AMF) and above‐ground phenotypic plant traits, invertebrate communities, and intensity of herbivory between the populations and habitat types. We further examined if the composition of the root microbiota was linked to soil pH, plant nutrients and herbivory at the population level, and to individual plant traits indicating plant productivity and reproduction.

Our main research questions were as follows:

- How do *S. dulcamara* plant traits differ among populations or habitats? Because we expected to find strong abiotic and biotic differences among habitat types (*e.g*., in humidity, soil nutrients and invertebrate communities), the first question served to investigate how these differences were related to phenotypic differences in plant performance.
- Is the root microbiota of *S. dulcamara* structured by habitat type and environmental conditions? In terms of microbial richness, we expected the wetter, more nutrient‐rich environments (forest and rural) to host richer and more diverse microbial communities than beach or urban environments (Hartman & Tringe 2019; Islam *et al*. 2020). To separate the impact of plant *versus* environment on the microbiome, we also compared the microbial root communities of *S. dulcamara* to root microbiomes of a neighbouring plant species growing in the same environment.
- Is the composition of the root microbiota connected to plant performance traits across populations and habitats? Given that the composition of root microbiota can affect plant performance traits, the association may be stronger for beneficial microbial communities, such as AMF (Begum *et al*. 2019) in more stressed environments.

## MATERIALS AND METHODS

### Classification of populations into four habitats

Eight populations of *S. dulcamara* were identified in southern Sweden within approximately 50 km of each other and categorized into four different pairs based on what type of habitat they were found in: *beach, forest, rural*, and *urban* (Fig. 1, see Field site descriptions in Text S1). Beach and urban habitats were ‘dry’ and forest and rural habitats were ‘wet’ (Fig. 1c). Plants in wet habitats experienced seasonal flooding with root systems submerged in water. Beach, rural, and urban habitats were ‘exposed’, while forest habitat was ‘shaded’ (50–296 *versus* 1.9–101 × 10 μmol m^−2^ s^−1^, respectively), measured as photosynthetically active radiation using a PAR quantum sensor (model SKP 215, Skye Instruments Ltd., United Kingdom) (Fig. 1c, Table S1). The two urban populations were situated in the cities of Malmö (population size circa 360 k people) and Lund (population size circa 130 k people), growing in small urban green spaces close to impervious surface. Based on the population spread (50–400 m) and approximate number of shoots (<100 to >10,000) (Table S1), the two beach populations were large, the two urban populations were small, while the populations of the other habitats were variable in size (Fig. 1c).

Indicator and trait values listed for *S. dulcamara* by Tyler *et al*. (2007) include ‘wet’ as its *moisture* niche, *light* optimum of ‘half‐shade–moderate shade’, and a sub‐neutral–circumneutral pH niche (~ pH 5.5–7.5). Our populations show a broader niche than expected regarding both moisture and light conditions (exposed–shaded, and wet–dry). pH (see Results) ranged between 5.7 and 8, which was slightly broader than expected.

### Population surveys of abiotic and biotic factors

To investigate differences among habitats and populations, we surveyed the abiotic factors temperature (°C) and relative humidity averages (air), soil properties and nutrients, and the biotic factors plant species richness (# spp) (Table S1) and the size and composition of invertebrate communities (Fig. 1b).

#### Temperature and relative humidity measures

Temperature (°C) and relative humidity averages were performed at the growth level of *S. dulcamara* plants between August 21st and October 8th 2020, using Tinytag probes (Tinytag plus 2 TGP4500, Gemini Data Loggers). The sampling dates represented the time from root microbiome sampling (see below) to the end of the growing season. Data were collected every 10 min. From this data, we calculated a daily mean temperature and daily mean relative humidity over the season.

#### Soil sampling and quantification of soil properties

To quantify population soil properties (soil clay, sand and organic matter content, pH, conductivity, mineral N content (N min), and plant available P, K, Mg, Ca) a W‐shaped transect of 10 soil cores (sampled to the depth of 0.25 m approximately 0.5 m apart where *S. dulcamara* was growing) was collected in August 2020 at the same time as root samples for microbiome analysis (see below). Surface debris was removed before sampling. Stones and plant material were removed from the samples, except for occasional fine root material. The samples were stored at −20 °C and then sent to Eurofins (https://www.eurofins.se) for soil profiling (Table S2). For the population in Lomma beach, Malmö and Lund, two transects of 10 cores were combined and analysed. The reason for this was as follows: The populations in Lomma beach was very large (>10,000 shoots) and spanned over a large distance (350 m), the Malmö population had asphalt below the soil so a reduced amount of soil was collected from each core, and in Lund, the population was spread over two different sampling points, approximately 400 m apart, so one transect was taken at each point and then combined for soil analysis.

#### Invertebrate sampling

To get an overview of how invertebrate communities (and the presence of potential antagonists and beneficials) varied between populations and habitats, we performed two types of invertebrate trap collections for a selection of the populations (at least one population from each habitat type – Kämpinge (B2), Alnarp pond (F1), Habo Ljung (F2), Borgeby (R1), Lund (U1)). We used white pan traps (500 ml Hammarplast bowl, 16 cm in diameter, 4.5 cm in depth) filled with water and a drop of detergent. We placed five traps per population with a minimum of 5 m apart (4 in Lund as there was limiting space) on the ground and pinned them down with tent pegs right next to *S. dulcamara* plants to target invertebrates that might interact with *S. dulcamara*. Pan traps are in general thought to efficiently catch non‐lepidopteran pollinators (*Hymenoptera, Diptera, Coleoptera*) and flies (*Diptera*), but also catches ground‐dwelling beetles (*Coleoptera: Carabidae* and *Staphylinidae*) and ants (*Hymenoptera*: *Formicidae*) (Montgomery *et al*. 2021). Traps were left out for 2 weeks (23 June 2020 – 7 July 2020) when invertebrates are highly active in Sweden. The traps were emptied once per week. In the second week, yellow sticky traps (Biobasiq, 20 × 25 cm) attached to bamboo sticks (approximately 0.5 m above‐ground) were put out as well (one per selected population); these were intended to catch flying insects. Trap catches were analysed under a dissecting microscope to quantify the number of individual invertebrates and classified to the taxonomic level order. Some specimens could only be identified to higher taxonomic levels, and other specimens belonged to orders that were rarely found in the samples and thus not possible to analyse statistically. These specimens were merged into the category ‘Other’ in statistical analyses.

### Plant measures across populations

To investigate how *S. dulcamara* differed among populations and habitats, as well as to study the link between plant performance and the root microbiome, we measured population herbivory, plant dry matter and nutrients, and a number of plant performance traits from shoots where we also collected material for root microbiome analysis (Fig. 1b).

#### Herbivory analysis

At the time of invertebrate collections (June–July 2020), leaves were collected from each population (five leaves from each of five individuals in each population) to evaluate herbivory pressure (Fig. 1b). Each leaf was pressed between two plexiglass sheets and photographed individually. The photographs were processed in ©Adobe Photoshop where leaf surface area and missing leaf surface area (holes and reconstructed edges if larger parts of the leaf had been eaten) were identified using select and mask tools and colour coded in blue (leaf) and black (holes) in order to use pixel counts to assess what percentage of the leaf surface had been eaten (see example pictures in Fig. S1). The images were then further processed in colab using a custom script (https://colab.research.google.com/drive/1t9JsYiT3W0jTfEHuH6WnSV3T9XmsP77M?hl=sv) to calculate what percentage of the leaf surface had been eaten (hereafter referred to as herbivory intensity).

#### Quantification of plant dry matter and nutrients

To quantify differences in plant nutrient status at the population level, above‐ground plant material from each population (collection sample with five top shoots from 10 to 15 places within each population) was collected at the same time as soil and root samples (August 2020), dried at 60 °C for a minimum of 24 h, and sent to Eurofins for plant nutrient profiling (N, P, K, Ca, Mg) Eurofins (https://www.eurofins.se) (Table S2). We measured both plant N and nitrate as the former gives an estimate of the nitrogen the plant has used for growth and development, and the latter, how much nitrogen the plant has stored (Fredes *et al*. 2019).

#### Plant performance measurements

Performance of *S. dulcamara* shoots of plants that we sampled for analysis of root microbiomes in August 2020 (see below) was also thoroughly examined and measured in the field (*n* = 12 per population (except for Malmö (U2), *n* = 10 due to small population size)). When plants were too big to sample as a whole (74%, see Table 1), we collected shoots from runners (as *S. dulcamara* propagates both vegetatively and sexually) by cutting off the runner 15 cm in each direction from the collected shoot. We ensured a minimum of 5 m between sampling spots to increase the chance of measuring different individuals. We recorded the following traits: number of shoots, total length of shoots, longest root, number of flowers, number of berries, and whether the shoot came from a runner from a large clone or was a smaller individual plant (as an indication of clone size or possibly plant age). We also recorded the presence or absence of holes in the plant leaves as a measure of herbivore exposure. All above‐ground material (including runners, fruits, etc.) was collected and dried at 60 °C for a minimum of 24 h, after which its dry weight was recorded as a measure of biomass.

**Table 1:** Mean (median) of plant traits across four habitats and eight populations of *Solanum dulcamara*.

dependent variable	independent variable	K‐W chi‐squared	df	*P*	pairwise comparisons (Dunn test, Bonferroni)							
					beach		forest		rural		urban	
					*Lomma beach*	*Kämpinge*	*Alnarp pond*	*Habo Ljung*	*Borgeby*	*Tvedöra*	*Lund*	*Malmö*
					B1	B2	F1	F2	R1	R2	U1	U2
Herbivory intensity (%)	Habitat	**81.8**	**3**	**<0.0001**	**1.1 (0.23)**		**4.6 (3.3)**		**9.7 (5.1)**		**0.67 (0.36)**	
					**b**		**a**		**a**		**b**	
	Population	**121**	**7**	**<0.0001**	**0.14 (0)**	**2.1 (1.2)**	**1.1 (0.72)**	**8.1 (6.6)**	**11.8 (5.4)**	**7.5 (3.6)**	**0.86 (0.48)**	**0.49 (0.32)**
					**b**	**c**	**bc**	**a**	**a**	**a**	**bc**	**bc**
Number of shoots	Habitat	7.27	3	0.06	2.3 (2.0)		1.7 (1.0)		1.3 (1.0)		2.1 (1.5)	
	Population	**24.7**	**7**	**0.0009**	**1.3 (1.0)**	**3.3 (3.0)**	**1.9 (1.0)**	**1.4 (1.0)**	**1.2 (1.0)**	**1.5 (1.0)**	**1.8 (1.0)**	**2.5 (3.0)**
					**b**	**a**	**ab**	**b**	**b**	**ab**	**ab**	**ab**
Combined length all shoots (cm)	Habitat	**14.7**	**3**	**0.002**	**102.6 (85.5)**		**110 (102)**		**157.1 (159)**		**169.1 (129)**	
					**b**		**b**		**a**		**ab**	
	Population	**21.5**	**7**	**0.003**	**78.1 (73.0)**	**127 (115)**	**110 (107)**	**110 (82.5)**	**182 (178)**	**132 (135)**	**138 (132)**	**207 (121)**
					**a**	**ab**	**ab**	**ab**	**b**	**ab**	**ab**	**ab**
Dry weight above‐ground biomass (g)	Habitat	**18.5**	**3**	**0.0003**	**19.2 (13.8)**		**8.82 (5.27)**		**13.24 (10.5)**		**30.66 (19.5)**	
					**a**		**b**		**ab**		**a**	
	Population	**21.4**	**7**	**0.003**	**15.5 (10.3)**	**22.8 (17.5)**	**8.88 (6.77)**	**8.78 (4.60)**	**15.3 (14.9)**	**11.1 (8.73)**	**27.0 (18.8)**	**35.1 (25.2)**
					**ab**	**ab**	**ab**	**b**	**ab**	**ab**	**ab**	**a**
Longest root (cm)	Habitat	**19.5**	**3**	**0.0002**	**20.8 (14.8)**		**40.6 (33.8)**		**23.5 (21.0)**		**37.4 (30.5)**	
					**c**		**a**		**bc**		**ab**	
	Population	**36.7**	**7**	**<0.0001**	**12.2 (10.8)**	**29.5 (21.0)**	**50.0 (56.5)**	**31.2 (23.0)**	**18.3 (16.0)**	**28.2 (25.0)**	**42.2 (42.0)**	**31.6 (20.8)**
					**b**	**ab**	**a**	**ab**	**b**	**ab**	**a**	**ab**
Number of flowers	Habitat	6.74	3	0.08	6.5 (1.5)		3.5 (0)		2.3 (0)		1.5 (0)	
	Population	14.0	7	0.05	5.9 (1.5)	7.2 (3.0)	0.10 (0)	7.0 (1.5)	0.80 (0)	3.8 (0)	0.9 (0)	2.2 (0)
Number of berries	Habitat	**36.2**	**3**	**<0.0001**	**37.4 (14.0)**		**1.1 (0)**		**5.5 (4.0)**		**9.9 (5.5)**	
					**a**		**c**		**b**		**b**	
	Population	**45.4**	**7**	**<0.0001**	**34 (19)**	**41 (8.5)**	**0.50 (0)**	**1.7 (0)**	**5.9 (4.5)**	**5.2 (1.5)**	**4.6 (0)**	**16 (17)**
					**a**	**ab**	**c**	**c**	**abc**	**abc**	**bc**	**ab**
Runner versus whole plant (n/total n)	Habitat				17/24		23/24		16/24		14/22	
	Population				11/12	6/12	11/12	12/12	5/12	11/12	6/12	8/10
Herbivory exposure (n/total n)	Habitat				18/24		24/24		24/24		20/22	
	Population				6/12	12/12	12/12	12/12	12/12	12/12	10/12	10/10

Herbivory intensity was estimated in June–July, while other performance traits and herbivory exposure were measured in plants sampled for root microbiome analysis in August. Differences among habitats and populations were tested using Kruskal–Wallis tests and Dunn tests, Bonferroni. Different letters indicate significant (*P* < 0.05) differences. Significant differences are shown in bold.

### Collection of root samples for microbiome analysis

Root samples were collected in August 2020 over 1.5 weeks. From each population, the following samples were collected for sequencing: *n* = 12 root samples from *S. dulcamara* (*n* = 10 from Malmö, U2), *n* = 4 root samples from another herbaceous species and *n* = 1 soil sample (soil core) taken close by the *S. dulcamara* plants. We collected roots from a neighbouring plant to get an indication of the influence of the plant *versus* environment on the microbiome in our *S. dulcamara* populations. Because of the large differences between the investigated habitats, we used in total four other species, and in most cases, it was possible to find the same species in the two populations in each of the four habitats. These additional species were: *Artemisia vulgaris* L. – Mugwort (beach: Lomma beach (B1) *n* = 4, Kämpinge (B2) *n* = 4, urban: Lund (U1) *n* = 2 Malmö (U2) *n* = 4, *Lycopus europaeus* L. – European water horehound (forest: Alnarp pond (F1) *n* = 4, Habo Ljung (F2) *n* = 4)), *Lysimachia vulgaris* L. – Yellow loosestrife (rural: Borgeby (R1) *n* = 4, Tvedöra (R2) *n* = 4), and *Plantago lanceolata* L. – Narrowleaf plantain (Lund (U1) *n* = 2) (Fig. 1). For the Lund population, we sampled the only *A. vulgaris* found and complemented this with two samples of *P. lanceolata*.

Collecting roots for microbiome sampling (from *S. dulcamara* recorded for performance traits, see above or from the additional species), we took care to trace all roots back to the main plant. When needed, a sterilized spade was used for digging up roots. Plant roots were processed immediately in the field using a field lab set‐up with a portable lab bench (IKEA klipsk, foldable bed tray, Fig. 1b). In all populations, it was possible to dig up the root systems without much surrounding soil, sand, or water sludge adhering to the roots. Young (not dead or woody) roots were cut off from the runner or stem with a sterilized pair of scissors and handled with sterilized forceps. The roots were placed in a 50 ml Falcon tube with 25 ml autoclaved MilliQ water and shaken vigorously for 30 s to remove soil particles (even roots without soil were cleaned in this manner for consistency). The roots were then removed from the water with sterilized forceps and dabbed on a filter paper in a petri dish to dry off excess liquids, and placed in a new tube for downstream analysis. All root samples were kept on ice in a portable freezer in the field and then stored in a − 20 °C freezer until DNA extractions were performed.

### Root processing, DNA extractions, and microbiome sequencing

Root samples were ground in liquid nitrogen, and for each plant, 230–250 mg of the homogenized root material was used for DNA extractions using the DNEasy PowerSoil Pro kit (Qiagen) following the manufacturer's protocol. Blank controls were included from the kit as well as a reference sample of a mock community (ZymoBIOMICS™ Microbial community standard II) with a known microbial composition to control for contamination or sequencing issues. Samples were sent for downstream analysis (Quality control), PCR and Illumina amplicon sequencing with primers targeting bacteria (16S: 799F & 1193R), general fungi (ITS2: ITS3‐2024F & ITS4‐2409R), and AMF (NS31F & AML2R) at Novogene.

### Statistical analyses

We analysed habitat differences among populations with SPSS (IBM Corp, 2023) using non‐parametric Kruskal–Wallis tests for temperature and relative humidity, and one‐way ANOVAs for plant species richness, soil properties, and plant dry matter and nutrients. Moreover, multivariate analyses of habitat differences were performed for all soil properties and plant dry matter and nutrients, respectively.

Statistical analyses of invertebrate diversity were carried out using Jmp version 17 (SAS Institute, 2023). We analysed the difference in number of invertebrates within each trapped order (*Aranae, Coleoptera, Diptera, Hymenoptera, Hemiptera* for the pan‐trapped samples and *Hymenoptera, Hemiptera, Coleoptera, Diptera* for the sticky trapped samples) as well as *Other* and the *Total number of collected invertebrates* for both trap types, depending on moisture of the habitat, using generalized linear models with a Poisson distributed response variable and logit link function. When needed, we controlled for overdispersion by using a generalized regression model with the negative binomial distribution of the response variable. Humidity was included in all the models as an explanatory variable (wet or dry). For the pan‐trapped invertebrates, we also performed separate models with population as explanatory variables; this was, however, not possible for sticky‐trapped data due to the sample size.

Statistical analysis of differences among habitats and population in herbivory intensity and plant performance traits was performed in R studio using the packages: vegan (Oksanen *et al*. 2025), moments (Komsta & Novomestky 2022), ggpubr (Kassambara 2023), FSA (Ogle *et al*. 2025), rcompanion (Mangiafico 2025) and ggplot2 (Wickham 2016). Due to a skewed distribution of this data, we used non‐parametric Kruskal–Wallis and Dunn tests.

To investigate the potential relationship between invertebrate diversity and herbivory, we used regression analyses for each invertebrate group and the herbivory intensity in each of the tested five populations. We performed one regression for each invertebrate group from the pan traps (*Aranae, Coleoptera, Diptera, Hymenoptera, Hemiptera*) as well as for *Other* and the *Total number of collected invertebrates*, and one for each invertebrate group from the sticky traps (*Hymenoptera, Hemiptera, Coleoptera, Diptera*) as well as for *Other* and the *Total number of collected invertebrates* with this trap type.

Visualization of differences among populations and habitats in soil properties and plant nutrients and performance traits, respectively, was performed with principal component analysis (PCA) using packages vegan, devtools (Wickham 2016), ggbiplot (Vu 2024), ggplot2 (Wickham 2016), ggfortify (Horikoshi & Tang 2018), gridExtra (Auguie & Antonov 2017), carData (Fox *et al*. 2022), car (Fox & Weisberg 2019), factoextra (Kassambara & Mundt 2020) and corrplot (Wei & Simko 2024) in R studio (R version 4.4.2, R Core team, 2024).

### Bioinformatics and statistics for microbiome data

Sequencing data were processed in R through the DADA2 pipeline (Callahan *et al*. 2016) identifying amplicon sequence variants (ASVs) (Callahan *et al*. 2017) of bacteria (based on the 16S reference database: silva_nr99_v138.1; Quast *et al*. 2013), general fungi (based on the UNITE general FASTA release for eukaryotes 2. Version 10.05.2021; Abarenkov *et al*. 2021), and AMF (MaarjAM database; Öpik *et al*. 2010). For the general fungal sequencing data, the primer pair used to amplify the ITS2 region (ITS3‐2024F & ITS4‐2409R) generated a relatively high amount of amplified plant DNA from our root samples, which was identified through the UNITE database for eukaryotes and subsequently removed before further analysis.

Statistical analyses of the bacterial and fungal sequencing data were performed in RStudio (R version 2022.12.0) using the microeco package and incorporated analyses (Liu *et al*. 2021). Briefly, for alpha diversity (diversity within samples), we performed separate analyses to test for differences among (i) habitats, (ii) populations, and (iii) investigated plant species, respectively, using the Kruskal–Wallis rank sum Test; paired groups were compared with Wilcoxon rank sum tests and Dunn's Kruskal–Wallis multiple comparisons when pairs of groups were more than two. We present Chao1, as we wanted to assess differences that included rare species.

For beta diversity (community composition), Bray–Curtis dissimilarities were used to compare communities in samples, and separate PerMANOVAs (Anderson 2005) were performed to analyse differences in distances among (i) wet *versus* dry habitats, (ii) habitats, (iii) populations and (iv) investigated plant species, respectively, using the cal_manova function which was developed based on the adonis2 function of the vegan package (Oksanen *et al*. 2025) in R. Bray–Curtis distances were used together with selected environmental data from the populations and plant performance traits to visualize their associations using RDA analyses. We used a Mantel test to test for significant correlations between the Bray–Curtis distances and selected variables. Selected variables included soil pH, plant dry matter and nutrient properties measured at the population level, as the microbiota may support nutrient uptake, and plant performance traits. Because plant N and plant nitrate were strongly positively correlated (Pearson *R* = 0.86), we only included plant N in the analysis. There was also some collinearity among plant performance traits (Spearman *R* < 0.61). To reduce traits indicating vegetative growth, we excluded ‘number of shoots’ from the analysis, as it correlated with both shoot length, biomass, and number of berries.

## RESULTS

### Soil profiles differed across habitats

The four habitats represented by our eight *S. dulcamara* populations did not differ in mean temperature (Kruskal–Wallis = 0.408, N = 7, df = 3, *P* = 0.41) or relative humidity (Kruskal–Wallis = 0.214, N = 7, df = 3, *P* = 0.98) estimated from late August to the beginning of October, or in plant species richness (F_3,4_ = 0.712, *P* = 0.59) (Table S1).

**Fig. 2:**
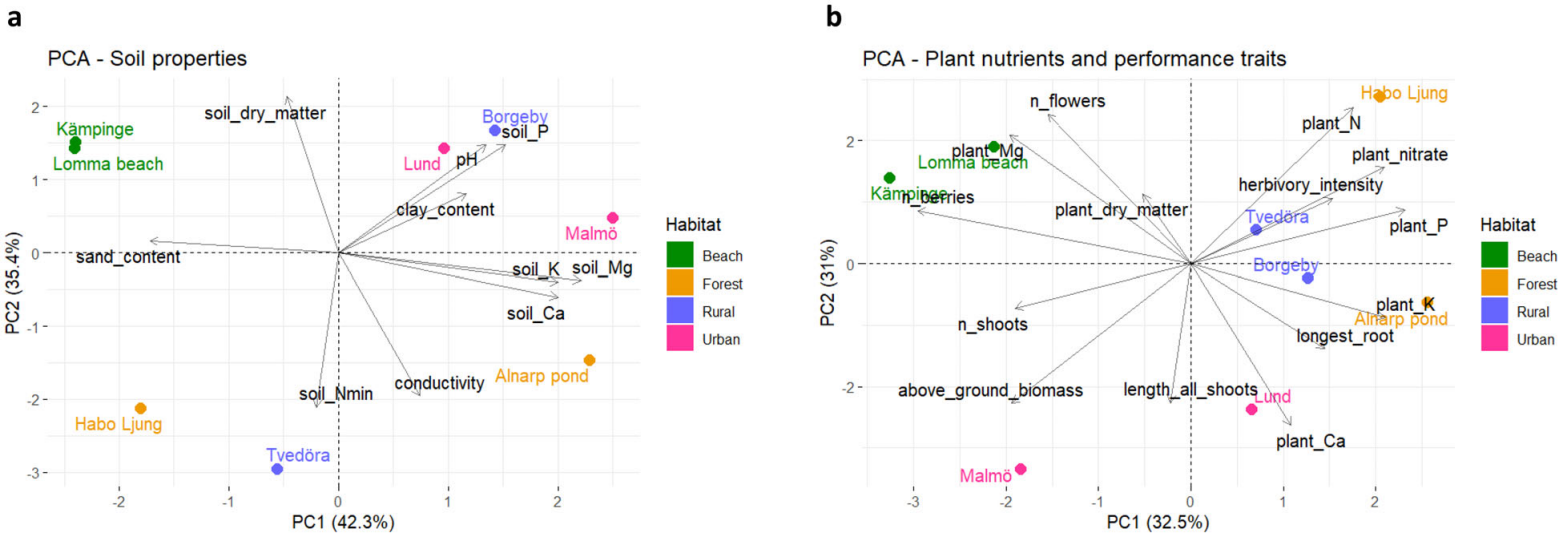
PCA Biplot visualizing the association between (a) soil properties and (b) plant dry matter, nutrients, and performance traits for eight *Solanum dulcamara* populations across four habitats. Plant dry matter and nutrients were estimated from top shoots. We used mean values for herbivory intensity and plant performance traits (see Table 1).

Analysis of 10 soil properties from the eight populations (one per population) collected in August (Table S3) showed an overall significant difference among habitats (Multivariate ANOVA (Willks' Lambda); F_12,2.94_ = 55.2, *P* = 0.04). A PCA Biplot visualizing the association between the soil properties and populations/habitats showed that in particular, the beach populations grouped together (Fig. 2a). The only significant soil factor among habitats was sand content, which was higher in beach and forest populations compared with rural and urban populations (Table S3).

### Some invertebrate communities differed between wet and dry habitats

Invertebrates were trapped by pan traps and sticky traps, respectively, during June–July in five of the eight *S. dulcamara* populations, representing all four habitats, and determined to order (Fig. S2). Investigating whether invertebrate orders differed depending on habitat moisture, we found no significant differences for the pan‐trapped invertebrate collections except for a tendency to have more Hemiptera and Araneae in dry habitats (Table S4). Data from the sticky traps revealed significantly more insects overall in the wet habitats, specifically from the Coleoptera, Diptera, and Hymenoptera orders (Table S4).

### Herbivory intensity was associated with habitat moisture and invertebrate abundance

Results from the leaf analysis performed at the time of invertebrate collections in June–July, estimating herbivory intensity across all eight populations, revealed that the sampled wetter forest and rural populations were significantly more plagued by herbivores (1.1–11.5%) than the drier beach and urban populations (0.1–2.1%) (Table 1, Fig. 2b). The rural population R1, Borgeby, had the plants with the overall highest herbivory intensity (up to 26%, 33%, and 58%), possibly indicating herbivory by slugs.

Regression analysis of invertebrate abundance in relation to herbivory intensity revealed positive regressions for Coleoptera and the total number of insects trapped by the sticky traps, that is, that herbivory intensity increased with an increased number of invertebrates (Table S4).

### Plant nutrients differed between habitats

Analysis of plant dry matter and nutrient status in August showed significantly higher plant N in forest populations compared with urban populations (Table S3) and a similar trend was seen for plant nitrate (*P* = 0.051) (Fig. 2b). Moreover, beach populations had higher amounts of magnesium than populations from other habitats. An analysis of plant dry matter and the six estimated nutrient factors did not indicate an overall significant difference among habitats (Multivariate ANOVA (Willks' Lambda); F_12,2.94_ = 5.73, *P* = 0.091) (Table S3). Values of N, K, and Ca showed similar patterns for soil and plant material, while P and Mg showed a different pattern (Fig. S3).

### Plant performance traits differed across habitats, with larger plants at dry sites and higher berry production in beaches

Most plant performance traits differed among both habitats and populations, exceptions being number of shoots and the number of flowers (Table 1). Plants were generally larger in the beach and urban habitats and smaller in the forest habitat (Fig. 2b), but rural habitats showed the longest total shoot length. Below‐ground, root length did not appear to be determined by wet *versus* dry habitats, as plants from the forest habitat had the longest roots followed by plants from the urban habitats (Table 1). Berry production was particularly high in beach habitats (Table 1, Fig. 2b). In one of the urban populations (U1, Lund), we noted trimming of hedges, which likely reduced the number of berries. Herbivory exposure (presence *versus* absence of holes in the leaves) was present in all collected plants in six of the populations (Table 1). In one of the urban populations (U1, Lund), herbivory exposure was present in 2 out of 12 plants and 6 out of 12 plants in one of the beach populations (B1 Lomma beach). This result is consistent with a higher herbivory intensity in the forest and rural populations found in June–July (Fig. 2b).

### Root microbial communities were dominated by shared ASVs, with beach populations exhibiting the lowest diversity

**Fig. 3:**
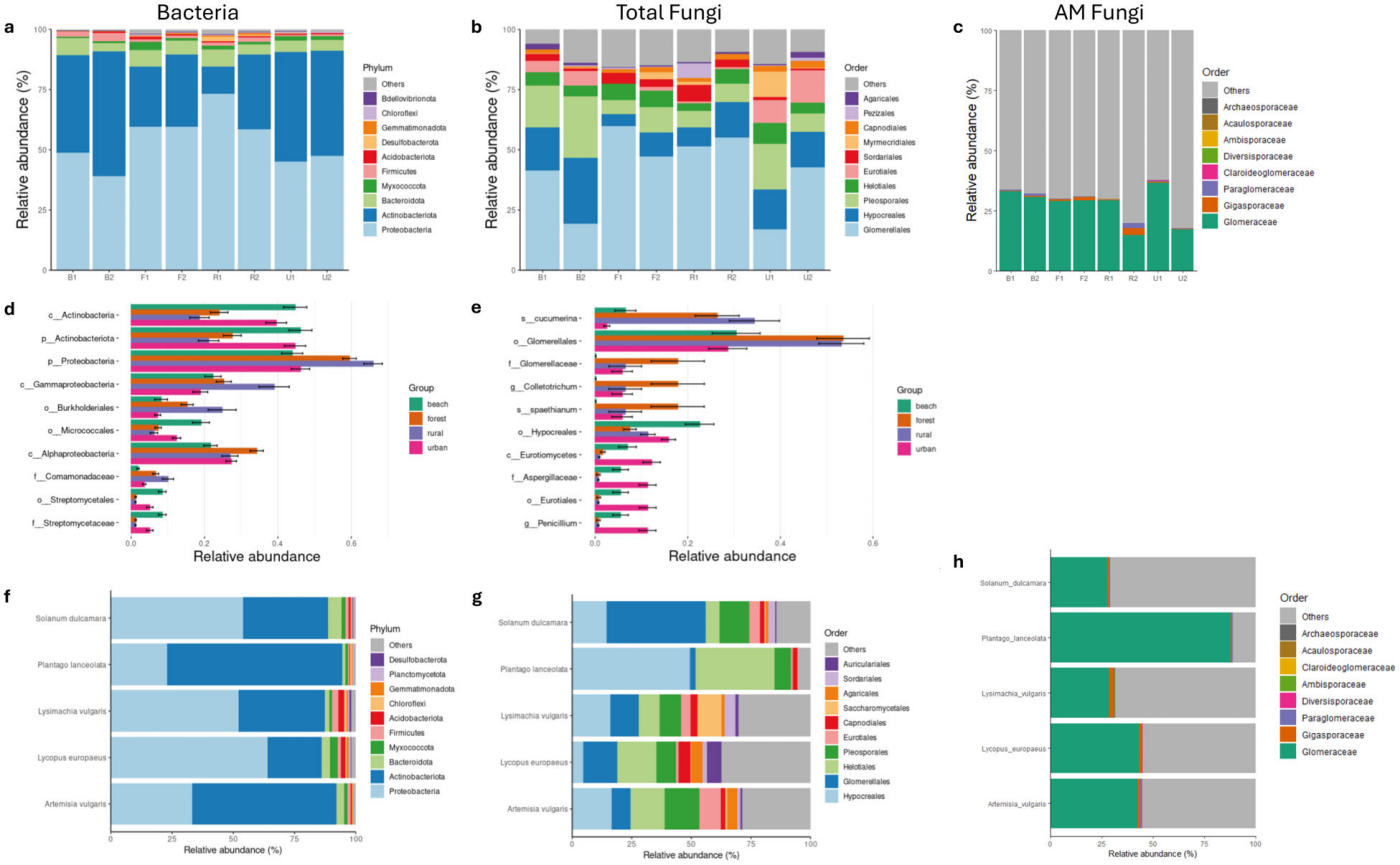
Differences in relative abundance (number of ASVs) of (a) bacterial phyla, (b) total fungal orders and (c) orders of AMF specifically, for root samples of *Solanum dulcamara* sampled across eight populations in four different habitat types (beach, B, forest, F, rural, R, urban, U). Bacterial communities are presented at the phylum level due to the high diversity of lower level taxa, whereas fungal communities (total fungi and AMF) are shown at the order level to better capture habitat‐ and population‐level differences. LDA‐scores from LeFSE analysis identifying the top 10 (g) bacterial and (h) total fungal phyla (p_), classes (c_), orders (o_), families (f_) or species (s_) driving the significant differences seen between *S. dulcamara* samples from the four habitats. Differences in relative abundance of (d) bacterial phyla, (e) total fungal orders and (f) AMF orders specifically, between root samples from the five plant species examined (*S. dulcamara n* = 94), *Plantago lanceolata n* = 2 (U1), *Lysimachia vulgaris n* = 8 (R1‐2), *Lycopus europaeus n* = 8 (F1‐2), *Artemisia vulgaris n* = 14 (B1‐2, U1‐2). 100% in each bar corresponds to the total microbial community detected in that sample after filtering.

To investigate a summary of sequencing statistics of the root microbiota of this study, including raw and filtered ASVs per sample for the different microbial groups (bacteria, total fungi, AMF) is given in Table S5. To investigate how the root microbiota of our eight *S. dulcamara* populations was structured across the four different habitat types, we first assessed compositional diversity (number of amplicon sequence variants, ASVs). Bacterial communities were dominated by Proteobacteria and Actinobacteriota at the phylum level (Fig. 3a). Looking at the top 10 most abundant taxa (Fig. 3d), community composition varied among habitats with Actinobacteria being relatively more abundant in the beach and urban populations, and Proteobacteria more abundant in the forest and rural populations. Alphaproteobacteria were more abundant in forest samples, and Gammaproteobacteria dominated rural samples (Fig. 3d).

Fungal communities associated with *S. dulcamara* were heavily dominated by Ascomyceta at the phylum level, and Basidiomyceta represented a comparatively minor fraction (data not shown). At the order level, compositional differences among habitats and populations became more pronounced. Across habitats, Glomerellales were the most abundant fungi (Fig. 3b), and this was the most abundant taxon in forest and rural habitats (Fig. 3e). Population‐level comparisons revealed enrichment of Pleosporales and Hypocreales in beach populations and in one urban (Lund, U1) population (Fig. 3b). Additionally, Eurotiales was more prevalent in beach and urban habitats, whereas Sordariales was more abundant in forest and rural habitats (Fig. 3b). Within the AMF dataset, Glomeraceae was the dominant order across habitats (Fig. 3c). However, most AMF were assigned to a diverse assemblage of low‐abundance taxa grouped as ‘others’ (Fig. 3c).

**Fig. 4:**
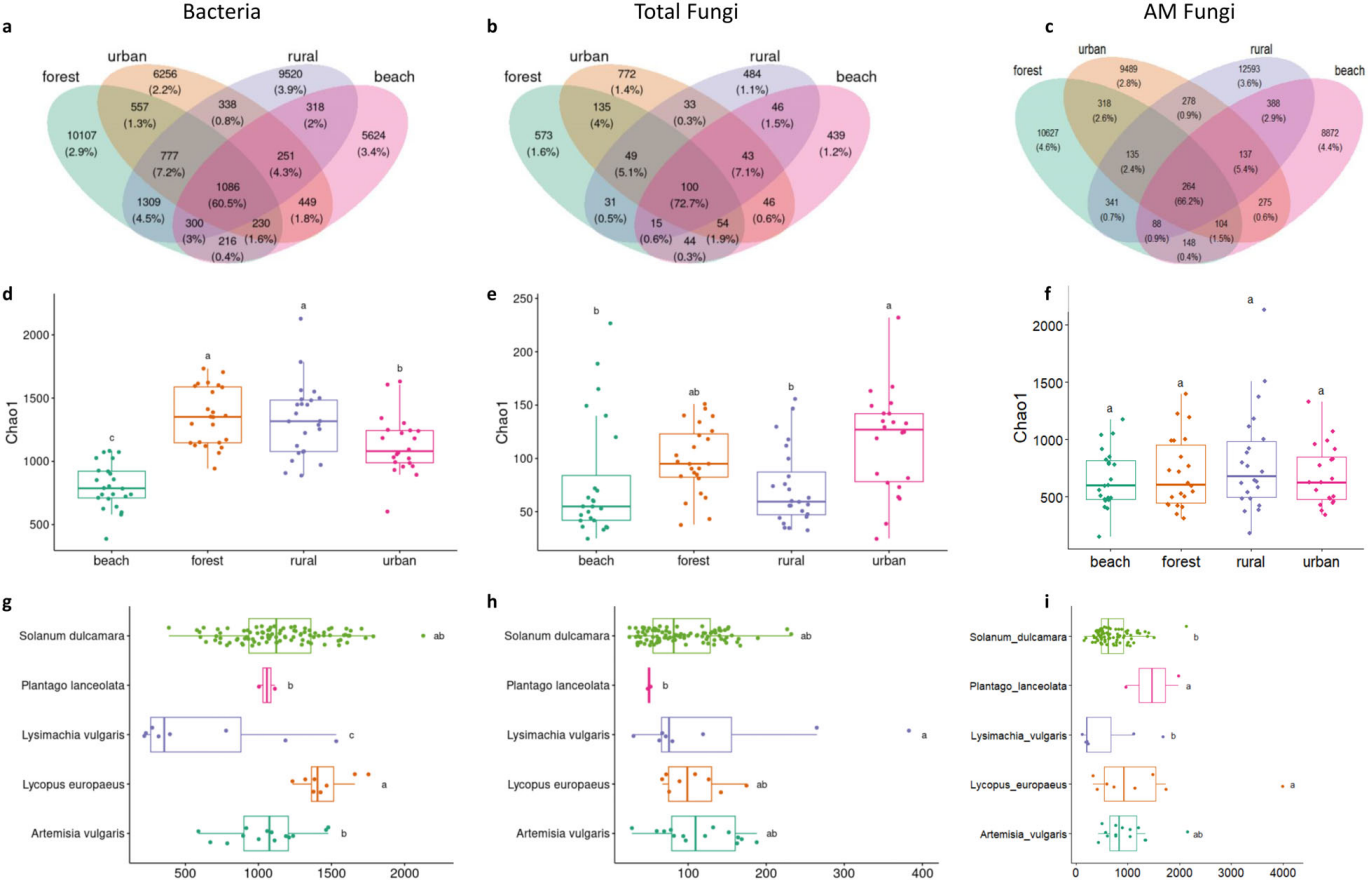
(a–c) Venn diagrams illustrating the amount of shared and unique ASVs for bacterial, total fungal, and AMF communities in root samples from eight *Solanum dulcamara* populations across four habitats. Values indicate the number of unique ASVs, and percentages represent the proportion of sequences associated with those ASVs relative to the total number of sequences. (d–f) Comparison of alpha diversity (Chao1) of bacterial, total fungal, and AMF communities in *S. dulcamara* roots across habitats. (g–i) Comparison of alpha diversity across the five plant species sampled in this study (*S*. *dulcamara n* = 94, *Plantago lanceolata n* = 2 (urban), *Lysimachia vulgaris n* = 8 (rural), *Lycopus europaeus n* = 8 (forest), *Artemisia vulgaris n* = 14 (beach and urban)).

Partitioning of ASVs across habitat types revealed consistent patterns in diversity and distribution. Beach habitats harboured the lowest sequence richness across all microbial groups (Fig. 4a–c). Most bacterial ASVs were shared across habitats, accounting for 60.5% of total sequences, although the forest populations had the highest number of unique variants (Fig. 4a). Similarly, 72.7% of fungal sequences were derived from a relatively small subset of shared ASVs (Fig. 4b). Although each habitat type contained a substantial number of unique fungal ASVs, these represented only a minor fraction of the total community (1.1–1.6%), with urban habitats harbouring the highest number of unique variants (Fig. 4b). In contrast, within the AMF dataset, rural populations exhibited the greatest number of unique ASVs, followed by forest, urban and beach populations (Fig. 4c). Overall AMF communities showed limited habitat‐specific differentiation, with shared ASVs accounting for 66.2% of the total sequences and with relatively few unique variants across habitats (Fig. 4c). The functional potential of both the major and the rare taxa is described in more detail in Text S2.

### Forest and rural habitats support higher bacterial diversity, while fungal patterns vary among populations

Patterns of within‐sample diversity (alpha diversity) revealed clear differences in the bacterial communities of *S. dulcamara* roots depending on habitat. Roots collected from wet habitats (forest and rural) harboured significantly higher bacterial diversity compared with those from dry habitats (beach and urban) (Fig. 4d). Among habitats, beach populations showed the lowest alpha diversity, whereas forest and rural populations exhibited the highest levels. Urban populations were intermediate (Fig. 4d). This trend was consistent when alpha diversity was assessed at the population level, where the same separation between wet and dry habitats was evident (Fig. S4a).

For fungal communities, total alpha diversity also varied significantly among habitats (Fig. 4e), although this pattern was not detected for AMF communities (Fig. 4f). Urban habitats displayed elevated total fungal diversity compared with the beach or rural populations (Fig. 4e). Pairwise comparisons suggested that the increased diversity was driven by the Malmö (U2) population, which overall had the highest values of alpha diversity across all populations (Fig. S4b).

### Beta diversity in the root microbiota among *S. dulcamara* habitats and populations

**Fig. 5:**
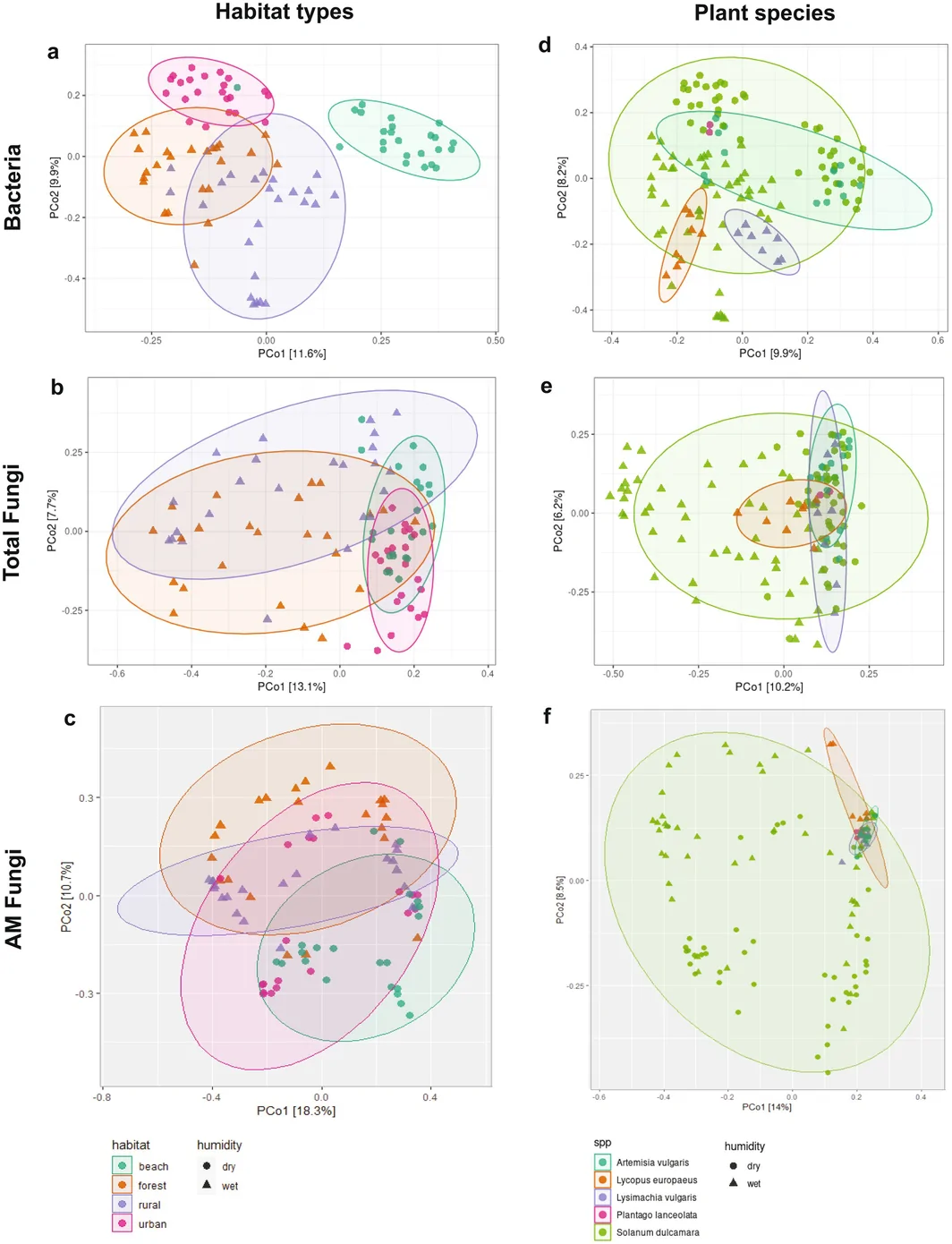
Principal coordinates analysis (PCoA) of Bray–Curtis distances between bacterial (a) total fungal (b) and AMF‐specific (c) communities in roots of *Solanum dulcamara* sampled in eight populations across four habitats and two humidity classifications (wet – triangles and dry – points). PerMANOVA results showed significant differences between dry and wet habitats and between all four habitats (Table S6,7). PCoA of Bray–Curtis distances between (d) bacterial, (e) fungal and (f) AMF communities in roots of the five different species sampled (*S. dulcamara n* = 94, *Plantago lanceolata n* = 2 (urban), *Lysimachia vulgaris n* = 8 (rural), *Lycopus europaeus n* = 8 (forest), *Artemisia vulgaris n* = 14 (beach, urban)). PerMANOVA results showed that *S. dulcamara* hosted significantly different communities compared with the other species (Table S6,7).

When comparing between‐sample diversity (beta diversity), measured as Bray–Curtis dissimilarities, bacterial communities clustered strongly by wet or dry habitats (Fig. 5a, Table S6) and differences were also detected among all four habitats individually (*P* = 0.001). At a finer scale, population identity also influenced bacterial community composition (Tables S6 and S7).

Fungal communities mirrored these patterns in terms of both total fungal and AMF‐specific beta diversity (Bray–Curtis distances) among samples between wet and dry habitats as well as across the four habitat types (Fig. 5b,c, Table S6). Pairwise comparisons confirmed that all habitats were compositionally distinct (fungi *P* = 0.001 and AMF *P* < 0.006, respectively). Again, population identity explained an additional layer of variation in fungal beta diversity (Tables S6 and S7).

### *Solanum dulcamara* exhibits wider AMF beta diversity and unique fungal composition compared with co‐occurring plant species

To investigate how species‐specific the root microbiota was among habitats and populations, we compared the samples from *S. dulcamara* and samples from the other four different plant species examined (one or occasionally two per habitat type). At the phylum level of the bacterial communities, the two species growing in the drier habitats (beach and urban) had higher proportions of Actinobacteriota compared with the two species at the wetter habitats (Fig. 3f), which mirrored the results in the *S. dulcamara* populations although the difference between habitats was not as distinct for *S. dulcamara* (Fig. 3a). The relative abundance of total fungal orders in root samples across the five different plant species examined showed a trend that Glomerellales was more dominant in samples of *S. dulcamara* than in the other species (Fig. 3g).

Alpha diversity of *S. dulcamara* compared with the other four species only showed significantly lower bacterial communities for *Lys. vulgaris* (rural habitat) (Fig. 4g) and significantly higher AMF communities for *P. lanceolata* (urban habitat) and *Lyc. europaeus* (forest habitat) (Fig. 4i). Thus, the lower bacterial and higher total fungal alpha diversity seen in beach and urban habitats, respectively, for *S. dulcamara* (Fig. 4d,e) was not reflected in samples of other species collected in beach and urban habitats (*A. vulgaris* and *P. lanceolata*) (Fig. 4g,h).

For beta diversity, bacteria communities differed between *S. dulcamara* and the two species sampled in the forest and rural habitats (*Lyc. europaeus* and *Lys. vulgaris*), as well as for the species sampled in the beach and urban habitats (*A. vulgaris* and *P. lanceolata*) (Fig. 5d, Tables S6 and S7). Beta diversity of total fungal and AMF‐specific communities also differed between plant species (Table S6). In the AMF communities specifically, *S. dulcamara* showed a wider beta diversity compared with the other plant species (Fig. 5f). Pairwise comparisons between *S. dulcamara* and the other sampled species, respectively, showed significant differences for all species for total fungi (Fig. 5e) and specifically in AMF (Fig. 5f, Table S7).

### Soil pH and plant nutrients drive root bacterial and fungal communities, while AMF show weaker associations

**Fig. 6:**
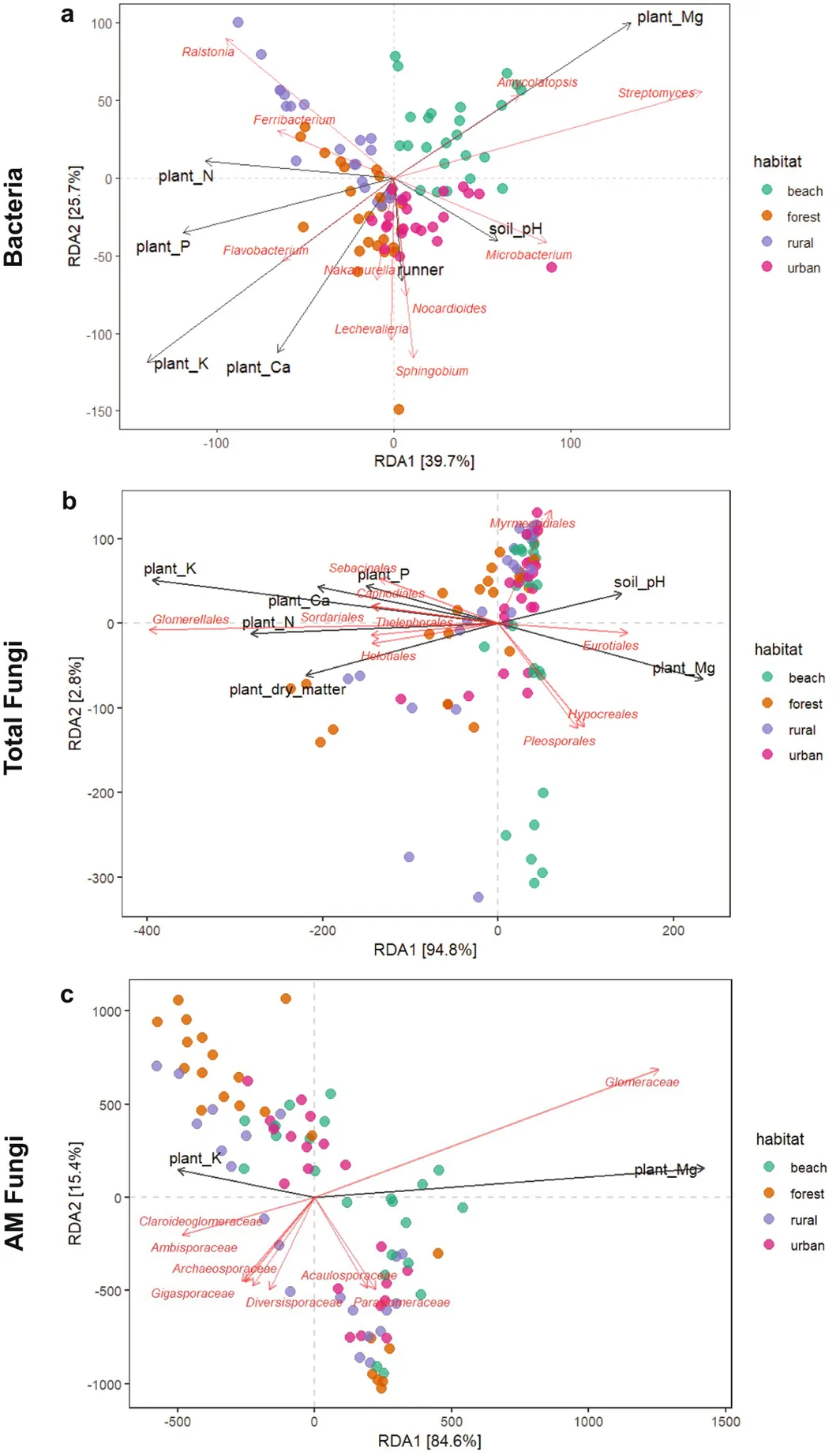
Redundancy analysis (RDA) of microbial communities based on (a) bacterial (genus level), (b) total fungal (order level), and (c) AMF (order level) specific sequences found in root samples of *Solanum dulcamara* sampled from eight populations and compared across four habitat types (*n* = 94). pH, plant dry matter, and nutrients at the population level, as well as plant performance traits from the same individuals sampled for root microbiota were correlated with the Bray–Curtis distance matrix using Mantel tests. The figures show significant (*P* < 0.05) variables.

The microbial community composition of bacteria and fungi correlated significantly with soil pH and the plant nutrients measured at the population level (Table S8). For AMF, we found no association with pH and only significant correlations with plant K and Mg, although there were non‐significant trends for correlations with plant N and Ca (*P* = 0.056 and 0.073, respectively). Plotting these data together in a distance‐based redundancy analysis (db‐RDA) suggested that the differences seen in bacterial and fungal communities between populations were associated with plant Mg in beach populations, plant P, K, and Ca in forest populations, plant N in both forest and rural populations, and soil pH in urban populations (Fig. 6a,b). For AMF, the two significant plant nutrients were similarly linked to habitat, but the distinction was less clear.

We found that most investigated plant performance traits and herbivory exposure collected from individual plants were uncorrelated with the composition of the bacterial, fungal, and AMF communities (Table S8). However, for bacteria there was an association with the status of the measured shoot, that is, whether it came from a runner or was an individual plant, as an indication of clone size.

## DISCUSSION

In this study on the wild, perennial vine *S. dulcamara*, we quantified differences in plant nutrients, herbivory, performance traits, and the root microbiota in eight populations across four distinct habitat types (beach, forest, rural, urban) within 50 km in southern Sweden. While the root bacteria and fungal community compositions were clearly distinct depending on habitat, we also identified a core set of microbes that were consistently present in the root microbiome across populations and habitats (61% of all bacterial and 73% of all fungal taxa). The microbiome was generally unrelated to herbivory and plant performance traits, but there were, however, relationships with both soil pH and plant nutrients at the population level.

### Plant phenotypic differences in herbivory and performance traits between habitats

The four investigated habitat types differed in overall soil properties, with a higher similarity among the dry *versus* the wet habitats. Moreover, the wet populations showed a higher number of invertebrates and more herbivory. In particular, the number of Coleoptera was positively linked to herbivory intensity, which corroborates previous studies that beetles are the main herbivores (Calf & van Dam 2012).

The eight populations also differed in plant nutrients and performance traits. As expected, the wet forest and rural habitats tended to host more nutrient‐rich plants, with significant differences in plant N between forest and urban habitats. Plants were generally larger and produced more berries in the dry beach and urban habitats. Other studies also found higher sexual reproduction relative to vegetative growth in perennials in drier habitats with lower nutrient levels and high light intensity, which may be explained by the need for increased competitive ability in terms of vegetative growth in resource‐rich environments (reviewed in Yang & Kim 2016). Below‐ground, the longest root was unrelated to the wet *versus* dry habitats, with the longest roots found in one wet forest population and one dry urban population. Extremely long roots could be favourable for exploiting a resource‐poor environment for access to water and nutrients, such as an urban environment, in line with the previously described ‘guerilla’ type growth (longer rhizomes and more spaced out ramets) in this species in response to a lack of resources (Cheplick *et al*. 1997). We confirm plant phenotypic differences among our eight investigated *S. dulcamara* populations and four habitat types.

### Impact of habitat type and environmental conditions on the root microbiota

Root systems of *S. dulcamara* showed distinct microbiota across habitats and populations, but the majority of the root microbiota was still made up of a relatively small core set that were omnipresent across habitats (60.5% for bacteria and 72.7% for fungi of all ASVs, respectively). These results are in line with the suggestion that the root microbiota consists of a ‘conserved core’ across environments and an ‘environment‐responsive sub‐community’ which may be unique for each environment (Vieira *et al*. 2020). The larger core microbiome seen for fungi is consistent with the hypothesis that fungal root microbiomes are more strongly influenced by the plant host than bacterial root microbiomes (Bergelson *et al*. 2019). The core microbiome found in *S. dulcamara* is higher than the 38% reported for the root microbiota in *A. thaliana* across 17 European populations from Sweden to Spain (Thiergart *et al*. 2020). Potentially, the difference may be explained by a smaller geographical and climatic gradient in our populations that occurred within about 50 km of each other.

Because it has been shown that environmental factors, including water, temperature, pH, and soil properties, can influence the soil and root microbiota (Hartman & Tringe 2019; Islam *et al*. 2020; Van Nuland *et al*. 2023), we expected to find differences among habitats. Growing under water can lead to oxygen deficiency and dry conditions can reduce the ability to take up nutrients and oxygen (Chen *et al*. 2023); therefore, we were particularly interested in differences between the wet (forest and rural) and the dry (beach and urban) habitats. We saw an overall lower bacterial alpha diversity (species richness and evenness) in beach and urban populations compared with the other populations. Bacterial beta diversity (community structure) was also distinct between dry and wet habitats, with a larger proportion of Gram‐positive Firmicutes in beach populations and more Gram‐negative Actinobacteria in forest and rural habitats. This result is consistent with a higher drought resistance in Gram‐positive bacteria compared with Gram‐negative bacteria (Schimel *et al*. 2007; Lennon *et al*. 2012). For fungi, there was no clear difference in alpha diversity between dry and wet habitats, but the urban populations had significantly higher diversity than the beach and rural populations. Microbial diversity has only been investigated across the urban–rural gradient in a few studies, with slightly contrasting results indicating both increased (Laforest‐Lapointe *et al*. 2017; Delgado‐Baquerizo *et al*. 2021) and decreased (Abrego *et al*. 2020) microbial diversity in urban areas. For AMF, that is, fungi known to be beneficial for plant nutrient and water uptake (Smith & Read 2008; Kakouridis *et al*. 2022), there was no difference in alpha diversity among habitats. This may imply an overall lower proportion of AMF of total fungi in urban areas, which is in line with the suggestion of a negative impact of urbanization on mutualistic relationships between plants and microbes (Whitehead *et al*. 2022; Khalid *et al*. 2024). Similar to bacteria, beta diversity for both general fungi and AMF showed a separation between the dry and wet habitats. Moreover, we found effects of local soil pH and of all measured plant nutrients on the composition of both the bacterial and total fungal communities in our study, suggesting that the local soil conditions are important at least when populations are sampled over a small geographic range. For AMF, only the plant nutrients K and Mg were linked to the community structure, possibly indicating a lower importance of soil conditions for the AMF community structure in *S. dulcamara* roots. In other wild species—A. *thaliana* and wild strawberry—sampled over a large geographical range, climatic and temperature differences had often larger effects than the soil conditions (Thiergart *et al*. 2020; Mittelstrass *et al*. 2021).

### Species‐specificity of root microbiota across habitats

To further investigate the extent to which the plant microbiota is determined by the plant or external factors (Dastogeer *et al*. 2020), we compared *S. dulcamara* to one or occasionally two neighbouring plant species for each population. Only a few differences in alpha diversity were detected between *S. dulcamara* and the other species. However, these differences did not reflect the differences in alpha diversity seen among *S. dulcamara* habitats. Beta diversity differed between *S. dulcamara* and the other species. These results indicate that at least part of the *S. dulcamara* microbiome is determined by the plant itself, for example, by the recruitment of beneficial microbes (Liu *et al*. 2021; Santoyo 2022; Hartman *et al*. 2023). Our analysis of the potential ecological functions of the taxa found in *S. dulcamara* roots showed a clear link between the habitat differences in moisture and relative occurrence of taxa adapted to such habitats, but also the presence of potentially mutualistic taxa (see Text S2). In other wild species, the host genotype is known to influence the bacterial and fungal root microbiome under field conditions (Ji *et al*. 2023; Wei & Tan 2023; He *et al*. 2024), suggesting differences among species in how much the microbial composition is shaped by the host *versus* the environment (Igwe & Vannette 2019; Maldonado *et al*. 2022). In *A. thaliana*, a species that does not harbour AMF, it appears that the host genotype has a minimal effect on the bacteria root microbiome (Thiergart *et al*. 2020). In future studies of *S. dulcamara*, it would be of interest to perform transplant experiments between habitats to get a better indication of the links between the microbiome, the host, and the environment.

### Relationship between plant performance traits and the root microbiota

Because of the growing number of studies that suggest a relationship between the microbiome and plant health, including herbivory (Pineda *et al*. 2010; Wang *et al*. 2023), we related our plant performance data to the composition of the root microbiomes. The associations found between the microbial community composition and plant nutritional status at the population level in *S. dulcamara* suggest that the microbiota has some influence on nutrient uptake in the plant, for example, by making nutrients more bioavailable to the plant (reviewed in Trivedi *et al*. 2020). However, most performance traits measured in the sampled plants (*e.g*., plant size and root length measures, number of berries and herbivory exposure) were uncorrelated to the composition of the bacterial or fungal communities. One exception for the bacterial composition was a correlation with the status of the sampled shoot (from a runner of a larger clone or from a smaller individual plant). This result may imply that recruitment may change over the plant life, given that larger clones are older plants, or that vertical transmission of microbes increase in larger clones (Vannier *et al*. 2019). For AMF, no associations were significant but there was a trend of a link with number of berries. The lack of any clear associations with plant performance at the individual shoot level, may imply that either microbial communities only have small effects on plant performance in *S. dulcamara*, or that the large phenotypic differences seen between habitats are masking the influence of the microbiome on these traits. It is also possible that we did not measure traits most relevant for plant performance. For example, our shoot measures may be a poor representation of plant performance in a clonal plant with unknown variation in size. We could also have estimated the percentage of herbivory in the sampled shoots as well as the fine root traits, as those can affect the assembly and composition of the root microbiota (Pérez‐Jaramillo *et al*. 2017; Spitzer *et al*. 2020). Moreover, our results may be influenced by the timing and frequency of our sampling, as the microbial communities could change over time (Han *et al*. 2023). Allocation trade‐offs between performance traits may also occur over years in perennial species (Journé *et al*. 2023), which may reduce our ability to detect correlations if for example individuals differ in resource availability (Van Noordwijk & De Jong 1986). Alternatively, we hypothesize that collectively, the microbial species present help the plant to optimize their phenotype to each of the environments (Hou *et al*. 2021; Mesny *et al*. 2023), which may be why there were no strong general patterns among samples. The potential role of the root microbiota in supporting plant phenotypic plasticity would be highly interesting to study in this species in the future.

## CONCLUSIONS

In this study, the root microbiome differed between four widely contrasting habitats of the wild perennial vine *S. dulcamara*, in particular in relation to dry *versus* wet habitats, in southern Sweden. However, more than 60% of the microbiome was similar across habitats, suggesting a large core microbial community. Although the microbial community composition was correlated to soil pH and plant nutrients measured at the population level, there were no clear associations with plant performance, which was contrary to our expectations. In future studies, it would be interesting to learn more about what determines the distinct root microbiome in this species and if it may be related to the capacity of this species to grow across extreme differences in habitats. Our study also provides one of the first insights into the root microbiome of *wild* urban plants and a comparison of how these differ from plants of the same species in rural areas. The impact of human activities on plant‐associated microbiota is still poorly understood but of importance in order to secure ecosystem stability and ultimately planetary health (Berg & Cernava 2022).

## AUTHOR CONTRIBUTIONS

The design of the experiment was conceived by KA and ÅL with critical input from LGB. Field sampling of plants, soil, and insects, as well as plant phenotyping, was carried out by KA, ÅL and assistance from CBA; processing of root samples and DNA extractions was done by KA. Bioinformatics and statistics for microbiome data were done by KA and NR with assistance from CBA (analysis of AMF sequences). Plant trait data were analysed by KA, and statistics for soil data were done by ÅL. A method and custom script for analysing herbivory intensity based on leaf photographs was provided by DK and then executed and statistically analysed by KA. KKG helped plan and organize insect sampling and processed and analysed the insect data. The writing of the manuscript was led by KA, ÅL, NR, and KKG, with critical input from LGB and edits from all authors.

## CONFLICT OF INTEREST

The authors declare no conflicts of interest.

## Supporting information


**Text S1.** Field site descriptions for *Solanum dulcamara* populations used in the study.
**Text S2**. Functional potential of major and rare taxa of root‐associated microbiota in *Solanum dulcamara*.
**Table S1**. Properties of *Solanum dulcamara* populations across four habitat types sampled in 2020.
**Table S2**. Methods used for estimation of soil and top shoot properties of *Solanum dulcamara* populations.
**Table S3**. Soil and top shoot nutrient properties analysed from the eight *Solanum dulcamara* populations across four habitat types. Differences between habitats were tested with one‐way ANOVAs for each variable (significant (*P* < 0.05) in bold). Different letters indicate significant differences in a *post hoc* test between habitat types. Soil nutrients P, K, Mg, and Ca were analysed in an air‐dried sample.
**Table S4**. Results from GLM of differences between wet and dry habitats in number of invertebrates and regression between herbivory intensity and number of invertebrates across sampled *Solanum dulcamara* populations. Separate analyses were made for invertebrates trapped with either pan or sticky traps, and for each collected invertebrate order, the group of small orders and other classes as well as the total number of invertebrates. No Aranae were found in the sticky trap collections. Significant (*P* < 0.05) results are highlighted in bold.
**Table S6**. Overview of results from perMANOVAs performed to compare beta diversity (Bray–Curtis distances) in the bacterial and fungal communities (including data from AMF‐specific primers analysed separately) in relation to four factors (Wet/dry habitat, Habitat, Population, Plant species). Habitat, population and wet/dry were compared between root samples of *Solanum dulcamara* whereas plant species was compared across all root samples. All analyses were run with 999 permutations.
**Table S7**. Pairwise comparisons from perMANOVAs performed to compare beta diversity (Bray–Curtis distances) in the bacterial and fungal communities (including data from AMF‐specific primers analysed separately) in relation to the factors Habitat, Population, and Plant species. Habitat and population were compared between root samples of *Solanum dulcamara* whereas plant species was compared across all root samples. All analyses were run with 999 permutations.
**Table S8**. Pearson correlations between selected environmental and plant performance trait variables and the Bray–Curtis distances for bacterial, fungal, and AMF communities, respectively, tested with Mantel tests. Soil pH, plant dry matter and nutrients were analysed per *Solanum dulcamara* population (*n* = 8) and plant performance traits measured in the plants sampled for root microbiome analysis in August (*n* = 94 from eight populations). Significant differences (*P* < 0.05) are shown in bold.
**Fig. S1**. Box plots from leaf analysis identifying the percentage of leaf surface areas missing (= eaten by herbivores) in (a) eight *Solanum dulcamara* populations and (b) across four habitats of these populations. (c) Five leaves were collected from five plants in each population and analysed using image analysis.
**Fig. S2**. Number of individual arthropods collected in five *Solanum dulcamara* populations with (a) sticky traps and (b) pan traps. Arthropods were classified to the taxonomic level order. The category Other combines specimens from various orders that were too few to statistically analyse on their own. Pan traps n: Alnarp pond (8), Borgeby (9), Lund (8), Habo Ljung (10), Kämpinge (9).
**Fig. S3**. PCA Biplot visualizing the association between soil and top shoot properties for eight *Solanum dulcamara* populations across four habitats.
**Fig. S4**. Box plots of alpha diversity (Chao 1) of (a) bacterial and (b) total fungal communities in *Solanum dulcamara* roots from eight populations and four types of habitats. Different letters indicate significant (*P* < 0.05) differences. Wet populations: A = Alnarp pond (F1), B = Habo Ljung (F2), D = Borgeby (R1), G = Tvedöra (R2); Dry populations: C = Lund (U1), E = Kämpinge (B2), F = Lomma beach (B1), H = Malmö (U2).

**Table S5.** A summary of sequencing statistics of samples of root microbiota of *Solanum dulcamara*, including total reads, raw and filtered ASVs per sample for the different microbial groups (bacteria, total fungi, AMF).

## Data Availability

All raw data sequences and scripts for bioinformatic analysis can be found in the following Github repository https://github.com/nataliar0312‐stack/Root‐Microbiomes‐S.‐dulcamara‐Across‐Contrasting‐Habitats‐. The taxonomic classification of all ASVs is available at Zenodo, https://doi.org/10.5281/zenodo.19454527. Other data is contained in the Swedish National Data Service (SND) Repository in Lankinen and Aleklett (2026), available from https://doi.org/10.5878/vfye‐4898.
