# Supplementary material for: Consistent root microbiomes across contrasting habitats in a wild perennial vine
Source: Plant Biol (Stuttg). 2026 Jul 6;28(6):1880–98. doi: 10.1111/plb.70245 (PMC13586648; doi:10.1111/plb.70245)
Supplement: Supplementary file 1 [file PLB-28-1880-s002.docx]

**Supporting Information**

**Text S1.** Field site descriptions for *S. dulcamara* populations used in the study.

*Beach*

*Lomma beach (B1):* This beach is situated along the waterfront in the small community of Lomma (population 13 648 ppl). The strip of beach is approximately 40 m wide, and sand dunes with vegetation separate the beach from a park-like recreational area featuring walking paths and managed lawns. The population of *S. dulcamara* grows in large streaks nestled in between the sand dunes and together with large patches of sand ryegrass (*Leymus arenarius*). These plants regularly get covered by sand from the dunes being blown around and occasionally from city management moving sand around on the beach with large bulldozers.

*Kämpinge (B2)*: This population grows in the intersection between a sandy beach and a shore meadow. It is 2.2 km away from more densely built-up areas, but there are pastures and a few farms in closer proximity. Some of the *S. dulcamara* plants grow down in the fine sand closer to the water, together with patches of *L. arenarius*, whereas other ones have formed larger bushes a bit further away from the sea in the more meadow-like strip of land.

*Forest*

*Alnarp pond (F1)*: This population grows along the shoreline of a small pond surrounded by large shading trees. The pond is situated in a peri-urban environment as it could be considered part of the extensive Alnarp park on the Alnarp campus of the Swedish University of Agricultural Sciences. It was historically used as a watering hole for horses from the nearby fire station. The surrounding vegetation gets managed periodically (eg. reeds get cut down, lawns get mowed) by park personnel. *S. dulcamara* grows out in the swampy edge of the pond and climbs among the reeds with roots and runners fully submerged.

*Habo Ljung (F2):* This population grows in an Alder marsh (alkärr) situated about 250 m inland from the sea. Light levels go from dark under the tree coverage to full sun in some spots further out in the reeds. The majority of plants have roots that are fully submerged under water, at least for parts of the year, even though it dries up a fair bit in the summer. The vegetation is dominated by reeds and Alder trees, but there is also a fair amount of sedge. *S. dulcamara* grows along the edge of the water and out among the reeds, climbing up the reeds for light.

*Rural*

*Borgeby (R1):* This population grows along a ditch in a small piece of land between two fields, one used as pasture and one used for intensive farming. Some of the *S. dulcamara* plants were growing up on the side banks of the ditch, and some were growing down in the water of the ditch. Soil was collected from the banks of the ditch. The surrounding vegetation was dominated by birch trees, reeds, tall grasses and forbs (eg. *L. vulgaris*).

*Tvedöra (R2)*: This population is situated inside a cow pen in a Salix marsh. All of the *S. dulcamara* plants here have root systems that are periodically fully submerged. The vegetation in this site is dominated by reeds and tall herbs, and surrounded by willows. The S*. dulcamara* plants are growing among the reeds with roots fully submerged in a waterlogged, sludge-like soil, growing dense mats of entangled roots.

*Urban*

*Lund (U1)*: The Lund population is situated within the campus area of Lund University. The *S. dulcamara* plants are growing inside and along the edge of planted Cotoneaster hedges at the side of a road and parking lot, as well as within a small courtyard for a university building. Throughout the growing season, the *S. dulcamara* plants produce shoots that grow out of the trimmed hedges and can easily be spotted, but as the hedges are managed, they also get regularly trimmed down together with the Cotoneaster. This part of the city has not been recently planted or developed, and there are records of this population dating back to at least the 1990s (Tyler, Johansson, Olsson & Sonesson, 2007).

*Malmö (U2):* The Malmö population grows on the side of a major road (~8.5 m from the road, directly in connection to a paved bike path) in a bushy strip of wild vegetation along a wire mesh fence. The immediately surrounding apartment buildings were recently built, and there are other ongoing construction sites nearby. The vegetation along the fence is dominated by climbing vines of *Clematis vitalba* cascading down to the pavement, but also has some larger individuals of *Salix caprea*.

**Text S2.** Functional potential of major and rare taxa of root-associated microbiota in *Solanum dulcamara*

Major taxa

Major bacterial taxa of the different habitats are outlined in Figure 3a,d. Actinobacteria that were relatively more abundant in beach and urban populations (Figure 3d), have been shown to **be adapted to oligotrophic and desiccation-prone conditions** (Barka *et al.* 2016). Proteobacteria, with a relatively higher frequency in the forest and rural populations, are associated with nutrient rich environments where they play a key role in soil C, N, and P cycles (Peng *et al.* 2025). Alphaproteobacteria, that had a high abundance in forest populations, are **commonly associated with nutrient cycling and organic matter turnover** (Poole *et al.* 2018). Gammaproteobacteria, found to dominate rural populations, are **typically considered copiotrophic** (Yue *et al.* 2023)

Major fungal taxa across habitats are shown in Figure 3b,e. The major fungal communities at the phylum level were dominated by Ascomycota**,** which are **groups broadly linked to decomposition, nutrient cycling, and soil structuring processes** (Tedersoo *et al.* 2014; Lindahl & Tunlid 2015). The order Glomerellales, **which include taxa with endophytic and pathogenic lifestyles** (Pérez-Pizá *et al.* 2023)**,** was the most common across habitats, and showed a relatively higher abundance in forest and rural habitas. The orders Pleosporales and Hypocreales, were more prevalent in beach populations and one urban population. While Pleosporales are known to dominate harsh conditions and semi-arid soil (Zhang [*et al.*](https://doi.org/) 2012), Hypocreales are typically associated with living hosts, including the plant endophytes Trichoderma (Harman *et al.* 2004). Eurotiales had also higher relative abundance in beach and urban habitats, **consistent with previous reports** (Samson *et al.* 2014). In contrast, in forest and rural habitats, Sordariales - **primarily saprobic fungi associated with organic matter decomposition - were more abundant.** For AMF, Glomeraceae **was generally the dominant order. Members of this group are well known for forming mutualistic associations with plant roots, enhancing nutrient uptake—particularly phosphorus—and contributing to soil health** (van Der Heijden *et al.* 2015).

Rare taxa

ASVs that were unique to each habitat were analysed based on their presence exclusively within a given habitat and absence from all others (data not shown). Bacterial community composition exhibited strong habitat-specific structuring, with each environment dominated by a distinct order, indicating pronounced environmental filtering of specialized taxa. Forest soils were overwhelmingly dominated by *Zavarziniales*, a lineage within the Alphaproteobacteria defined in genome-based taxonomies, belongs to a broader group of bacteria frequently associated with low-energy or oxygen-limited environments, suggesting adaptation to oligotrophic conditions (Cavellos *et al.* 2022). In rural habitats, *Burkholderiales* prevailed, consistent with their well-documented roles as plant-associated bacteria involved in nutrient cycling, rhizosphere interactions, and both mutualistic and pathogenic relationships (Tillmann *et al.* 2005; Compant *et al.* 2008). Urban environments were characterized by a high abundance of *Saccharimonadales* (Candidate Phyla Radiation), which are known for reduced genomes and dependence on host-associated or stressed environments, often linked to disturbed or anthropogenically influenced systems (Brown *et al.* 2015). In contrast, beach habitats were dominated by *Bacillales*, a group of spore-forming bacteria with high tolerance to environmental stressors such as salinity, desiccation, and UV radiation (Nicholson *et al.* 2000). Overall, these patterns highlight strong habitat filtering, whereby each environment selects for functionally adapted and habitat-specific bacterial groups.

Similarly, the ITS-based fungal community composition, restricted to habitat-specific ASVs, suggested potential differences in both taxonomic structure and inferred functional roles. Beach environments were strongly dominated by *Eurotiales*, which might indicate a selective system driven by environmental stressors such as salinity, desiccation, and UV exposure, favoring stress-tolerant saprotrophic taxa such as *Aspergillus* and *Penicillium* (Houbraken *et al.* 2020; Liu *et al.* 2023). In contrast, rural and urban habitats exhibited more even community distributions, with *Hypocreales*, *Pleosporales*, and *Helotiales* co-dominating, which could reflect a mixture of trophic strategies including saprotrophy, plant pathogenicity, and endophytism (Zhang *et al.* 2012; Glynou *et al.* 2018). The presence of *Saccharomycetales* in rural systems may be associated with nutrient-rich conditions associated with labile carbon inputs and agricultural management (Hannula *et al.* 2021), while the occurrence of orders such as *Onygenales* and *Pezizales* in urban environments could suggest an influence of disturbance and anthropogenic substrates (McGuire *et al.* 2013; Anane *et al.* 2015). Overall, these results are consistent with a gradient from highly selective, stress-driven fungal communities in beach environments to more functionally complex systems in forests, with rural and urban habitats occupying intermediate states.

AMF communities, also based on habitat-specific ASVs, showed a strong and consistent taxonomic dominance across habitats, with *Glomeraceae* overwhelmingly prevailing in forest, urban, and rural soils. This pattern is consistent with the well-documented ecological versatility of *Glomeraceae*, which are considered generalist and disturbance-tolerant AMF capable of rapidly colonizing roots and persisting under a wide range of environmental conditions (Stürmer & Kemmelmeier 2021).

**References**

Anane S., Al-Yasiri M.H.Y., Normand A.-C. & Ranque S. (2015) Distribution of keratinophilic fungi in soil across Tunisia: A descriptive study and review of the literature. *Mycopathologia* **180**, 61–68. https://doi.org/[10.1007/s11046-015-9870-9](https://doi.org/10.1007/s11046-015-9870-9)

Barka E.A., Vatsa P., Sanchez L., Gaveau-Vaillant N., Jacquard C., Klenk H.-P., … van Wezel G.P. (2016) Taxonomy, physiology, and natural products of Actinobacteria. *Microbiology and Molecular Biology Reviews* **80**, 1–43. <https://doi.org/10.1128/MMBR.00019-15>

Brown C.T., Hug L.A., Thomas B.C., Sharon I., Castelle C.J., Singh A., … Banfield J.F. (2015) Unusual biology across a group comprising more than 15% of domain Bacteria. *Nature* **523**, 208–211. <https://doi.org/10.1038/nature14486>

Cevallos M.A. & Degli Esposti M. (2022) New Alphaproteobacteria thrive in the depths of the ocean with oxygen gradient. *Microorganisms* **10**, 455. https://doi.org/10.3390/microorganisms10020455

Compant S., Nowak J., Coenye T., Clément C. & Ait Barka E. (2008) Diversity and occurrence of *Burkholderia* spp. in the natural environment. *FEMS Microbiology Reviews* **32**, 607–626. <https://doi.org/10.1111/j.1574-6976.2008.00113.x>

Glynou K., Nam B., Thines M. & Maciá-Vicente J.G. (2018) Facultative root-colonizing fungi dominate endophytic assemblages in roots of nonmycorrhizal *Microthlaspi* species. *New Phytologist* **217**, 1190–1202. <https://doi.org/10.1111/nph.14873>

Hannula S.E., Di Lonardo D.P., Christensen B.T., Crotty F.V., Elsen A., van Erp P.J., … Toth Z. (2021) Inconsistent effects of agricultural practices on soil fungal communities across 12 European long‐term experiments. *European Journal of Soil Science* **72**, 1902–1923. https://doi.org/10.1111/ejss.13090

Harman G.E., Howell C.R., Viterbo A., Chet I. & Lorito M. (2004) Trichoderma species -opportunistic, avirulent plant symbionts. *Nature Reviews Microbiology* **2**, 43–56. https://doi.org/[10.1038/nrmicro797](https://doi.org/10.1038/nrmicro797)

Houbraken J., Kocsubé S., Visagie C.M., Yilmaz N., Wang X.-C., Meijer M., … Frisvad J.C. (2020) Classification of *Aspergillus*, *Penicillium*, *Talaromyces* and related genera (Eurotiales): An overview of families, genera, subgenera, sections, series and species. *Genera of Hyphomycetes: a Tribute to Keith A. Seifert* **95**, 5–169. https://doi.org/10.1016/j.simyco.2020.05.002

Lindahl B.D. & Tunlid A. (2015) Ectomycorrhizal fungi - potential organic matter decomposers, yet not saprotrophs. *New Phytologist* **205**, 1443–1447. <https://doi.org/10.1111/nph.13201>

Liu C., Wang X.-C., Yu Z.-H., Zhuang W.-Y. & Zeng Z.-Q. (2023) Seven new species of *Eurotiales* (Ascomycota) isolated from tidal flat sediments in China. *Journal of Fungi* **9**, 960. <https://doi.org/10.3390/jof9100960>

McGuire K.L., Payne S.G., Palmer M.I., Gillikin C.M., Keefe D., Kim S.J., … Koshner J.A. (2013) Digging the New York City skyline: Soil fungal communities in green roofs and city parks. *PloS One* **8**, e58020. <https://doi.org/10.1371/journal.pone.0058020>

Nicholson W.L., Munakata N., Horneck G., Melosh H.J., & Setlow P. (2000) Resistance of *Bacillus* endospores to extreme terrestrial and extraterrestrial environments. *Microbiology and Molecular Biology Reviews* **64**, 548–572. <https://doi.org/10.1128/MMBR.64.3.548-572.2000>

Peng N., Wang Y., Wu H., Hao H., Sailike A., Yu Z., Li S., … Zhang, W. (2025). Phosphorus cycling dominates microbial regulation of synergistic carbon, nitrogen, and phosphorus gene dynamics during *Robinia pseudoacacia* restoration on the Loess Plateau. *Agronomy* **15**, 797. https://doi.org/10.3390/agronomy15040797

Pérez-Pizá M.C., Striker G.G. & Stenglein S.A. (2023) Seed-borne diseases in pasture grasses and legumes: State of the art and gaps in knowledge. *Journal of Plant Diseases and Protection* **130**, 225–244. https://doi.org/[10.1007/s41348-022-00703-7](https://doi.org/10.1007/s41348-022-00703-7)

Poole P., Ramachandran V. & Terpolilli J. (2018) Rhizobia: From saprophytes to endosymbionts. *Nature Reviews Microbiology* **16**, 291–303. <https://doi.org/10.1038/nrmicro.2017.171>

Samson R.A., Visagie C.M., Houbraken J., Hong S.-B., Hubka V., Klaassen C.H.W., … Frisvad J.C. (2014) Phylogeny, identification and nomenclature of the genus Aspergillus. *Studies in Mycology* **78**, 141–173. <https://doi.org/10.1016/j.simyco.2014.07.004>

Stürmer S.L. & Kemmelmeier K. (2021) The Glomeromycota in the neotropics. *Frontiers in microbiology* **11**, 553679. https://doi.org/10.3389/fmicb.2020.553679

Tedersoo L., Bahram M., Põlme S., Kõljalg U., Yorou N.S., Wijesundera R., … Suija A. (2014) Global diversity and geography of soil fungi. *Science* **346**, 1256688. <https://doi.org/10.1126/science.1256688>

Tillmann S., Strömpl C., Timmis K.N. & Abraham W.-R. (2005) Stable isotope probing reveals the dominant role of *Burkholderia* species in aerobic degradation of PCBs. *FEMS Microbiology Ecology* **52**, 207–217. https://doi.org/10.1016/j.femsec.2004.11.014

van Der Heijden M.G., Martin F.M., Selosse M. & Sanders I.R. (2015) Mycorrhizal ecology and evolution: The past, the present, and the future. *New Phytologist* **205**, 1406–1423. [https://doi.org/10.1111/nph.13288](https://doi.org/10.1111/nph.13288%20))

Yue Y., Gong X., Zheng Y., Tian P., Jiang Y., Zhang H. & Qi H. (2023) Organic material addition optimizes soil structure by enhancing copiotrophic bacterial abundances of nitrogen cycling microorganisms in Northeast China. *Agronomy* **13**, 2108. <https://doi.org/10.3390/agronomy13082108>

Zhang Y., Crous P.W., Schoch C.L. & Hyde K.D. (2012) Pleosporales. *Fungal Diversity* **53**, 1–221. https://doi.org/10.1007/s13225-011-0117-x

**Table S1.** Properties of *S. dulcamara* populations across four habitat types sampled in 2020.

| Population | Lomma beach | Kämpinge | Alnarp pond | Habo Ljung | Borgeby | Tvedöra | Lund | Malmö |
| --- | --- | --- | --- | --- | --- | --- | --- | --- |
|  | **B1** | **B2** | **F1** | **F2** | **R1** | **R2** | **U1** | **U2** |
| Habitat | *Beach* | | *Forest* | | *Rural* | | *Urban* | |
| GPS coordinates | N: 55.67007  E: 013.06250 | N: 55.390990  E: 013.003895 | N: 55.65854  E: 013.08458 | N: 55.68995  E: 013.05898 | N: 55.752266 E: 013.042667 | N: 55.71766  E: 013.43784 | N: 55.71194  E: 013.20644 | N: 55.589279  E: 012.939825 |
| Moisture condition | dry | dry | wet | wet | wet | wet | dry | dry |
| Light (x10µmol m^-2 s^-1) | 196 | 170 | 1.9 - 3.4 | 1.3-101 | 186 | 191 | 50 | 188 |
| Populations spread (m) | 350 | 400 | 100 | 150 | 50 | 180 | 400 | 80 |
| Approx. pop. size (# shoots) | >10 000 | 700-800 | <100 | ~2000 | <500 | >1000 | <150 | <500 |
| Mean temperature (^o^C)  21 Aug – 8 Oct | 16.7 | 16.0 | 16.5 | 14.8 | 15.4 | 14.2 | 16.2 | NA |
| Mean humidity (air)  21 Aug – 8 Oct | 80.8 | 87.5 | 82.3 | 89.9 | 84.0 | 86.1 | 85.2 | NA |
| Plant diversity (# spp) | 47 | 38 | 18 | 34 | 16 | 45 | 32 | 23 |

**Table S2.** Methods used for estimation of soil and top shoot properties of *S. dulcamara* populations.

|  | Analysis | Method | Measurement  uncertainty | Recognition |
| --- | --- | --- | --- | --- |
| **Soil properties** |  |  |  |  |
| Soil type |  | Nilsson, Lantbrukshöhskolan, Rapport 47, 1976 |  | ISO/IEC 17025:2017 SWEDAC 1912 |
| Clay content (%) |  | Internal method (In-House) |  | DIN EN ISO/IEC 17025:2018 DAkkS D-PL-20226-01-00 |
| Sand content (%) |  | DIN ISO 11277-2002-08 |  | DIN EN ISO/IEC 17025:2018 DAkkS D-PL-20226-01-00 |
| pH |  | ISO 10390: 2005-12 | ± 5.22 % | DIN EN ISO/IEC 17025:2018 DAkkS D-PL-20226-01-00 |
| Conductivity |  | SS EN 13038 |  | DIN EN ISO/IEC 17025:2018 DAkkS D-PL-20226-01-00 |
| Dry matter (%) |  | EN 15934: 2012-11 |  | DIN EN ISO/IEC 17025:2018 DAkkS D-PL-20226-01-00 |
| Nmin (kg/ha) |  | Internal method (In-House) |  | ISO/IEC 17025:2017 SWEDAC 1912 |
| P (mg/100g ) | P-AL | DIN EN ISO 11885:2009-09 | ± 8.02 % | DIN EN ISO/IEC 17025:2018 DAkkS D-PL-20226-01-00 |
| K (mg/100g) | K-AL | DIN EN ISO 11885:2009-09 | ± 23.38 % | DIN EN ISO/IEC 17025:2018 DAkkS D-PL-20226-01-00 |
| Ca (mg/100g) | Ca-AL | DIN EN ISO 11885:2009-09 | ± 17.39 % | DIN EN ISO/IEC 17025:2018 DAkkS D-PL-20226-01-00 |
| Mg (mg/100g) | Mg-AL | DIN EN ISO 11885:2009-09 | ± 16.62 % | DIN EN ISO/IEC 17025:2018 DAkkS D-PL-20226-01-00 |
| **Top shoot properties** |  |  |  |  |
| Dry matter (%) |  | Internal method (In-House) |  | BLGG AgroXpertus (Wageningen) |
| N (g/kg) |  | Internal method (In-House) |  | BLGG AgroXpertus (Wageningen) |
| nitrate (g/kg) |  | Internal method (In-House) |  | BLGG AgroXpertus (Wageningen) |
| P (g/kg) |  | Internal method (In-House) |  | BLGG AgroXpertus (Wageningen) |
| K (g/kg) |  | Internal method (In-House) |  | BLGG AgroXpertus (Wageningen) |
| Ca (g/kg) |  | Internal method (In-House) |  | BLGG AgroXpertus (Wageningen) |
| Mg (g/kg) |  | Internal method (In-House) |  | BLGG AgroXpertus (Wageningen) |

**Table S3.** Soil and top shoot nutrient properties analysed from the eight *S. dulcamara* populations across four habitat types. Differences between habitats were tested with one-way ANOVAs for each variable (significant (p < 0.05) in bold). Different letters indicate significant differences in a post hoc test between habitat types. Soil nutrients P, K, Mg and Ca were analysed in an air-dried sample.

| Habitat | *Beach* | | | *Forest* | | | *Rural* | | | *Urban* | | |  |
| --- | --- | --- | --- | --- | --- | --- | --- | --- | --- | --- | --- | --- | --- |
| **Population** | Lomma beach | Kämpinge |  | Alnarp pond | Habo Ljung |  | Borgeby | Tvedöra |  | Lund | Malmö |  |  |
|  | B1 | B2 |  | F1 | F2 |  | R1 | R2 |  | U1 | U2 |  | **p** |
| **Soil properties** |  |  |  |  |  |  |  |  |  |  |  |  |  |
| Clay content (%) | 2.0 | 2.0 |  | 6.0 | 2.8 |  | 16 | 3.6 |  | 10 | 2.8 |  | 0.55 |
| Sand content (%) | **98** | **98** | ***a*** | **77** | **86** | ***a*** | **53** | **68** | ***b*** | **69** | **71** | ***b*** | **0.015** |
| pH | 6.8 | 7 |  | 7.2 | 6.2 |  | 7 | 5.7 |  | 7.4 | 8 |  | 0.31 |
| Conductivity | 0.6 | 0.5 |  | 3.2 | 1.5 |  | 0.9 | 4.1 |  | 0.9 | 1.7 |  | 0.48 |
| Dry matter (%) | 95.9 | 98.5 |  | 44.1 | 32.4 |  | 70.8 | 34.2 |  | 89.5 | 70.5 |  | 0.56 |
| Nmin (kg/ha) | 20 | 18 |  | 259 | 467 |  | 66 | 325 |  | 63 | 62 |  | 0.13 |
| P (mg/100g ) | 6.3 | 5.3 |  | 7.8 | 2.6 |  | 14 | 3.1 |  | 8.9 | 9.4 |  | 0.75 |
| K (mg/100g) | 2.4 | 2.1 |  | 34 | 5.1 |  | 8.8 | 9.9 |  | 20 | 26 |  | 0.31 |
| Ca (mg/100g) | 28 | 41 |  | 1000 | 260 |  | 380 | 550 |  | 500 | 1300 |  | 0.30 |
| Mg (mg/100g) | 3.5 | 4.4 |  | 34 | 11 |  | 20 | 15 |  | 21 | 34 |  | 0.22 |
| **Top shoot properties** |  |  |  |  |  |  |  |  |  |  |  |  |  |
| Dry matter (%) | 90.8 | 91.7 |  | 91.1 | 91 |  | 91 | 92.6 |  | 88.4 | 91.4 |  | 0.55 |
| N (g/kg) | **33.8** | **33.8** | ***ab*** | **39** | **50** | ***a*** | **36.3** | **39.2** | ***ab*** | **26.5** | **22.2** | ***b*** | **0.039** |
| nitrate (g/kg) | 1.3 | 1.5 |  | 8.8 | 17.8 |  | 3.9 | 3.2 |  | 1.3 | 0.8 |  | 0.051 |
| P (g/kg) | 3 | 3 |  | 3.6 | 3.6 |  | 3.5 | 3.3 |  | 3.8 | 2.2 |  | 0.73 |
| K (g/kg) | 22.5 | 20.4 |  | 35.5 | 30.6 |  | 29.4 | 23.6 |  | 28.5 | 30.2 |  | 0.060 |
| Ca (g/kg) | 11.4 | 7.4 |  | 16.4 | 10 |  | 14.1 | 16 |  | 16.9 | 19.4 |  | 0.15 |
| Mg (g/kg) | **6.1** | **5.5** | ***a*** | **3.4** | **3.6** | ***b*** | **2.9** | **3.1** | ***b*** | **2.6** | **2.7** | ***b*** | **<0.001** |

**Table S4.** Results from GLM of differences between wet and dry habitats in number of invertebrates and regression between herbivory intensity and number of invertebrates across sampled *S. dulcamara* populations. Separate analyses were made for invertebrates trapped with either pan or sticky traps, and for each collected invertebrate order, the group of small orders and other classes as well as the total number of invertebrates. No Aranae were found in the sticky trap collections. Significant (p < 0.05) results are highlighted in bold.

| Trap type | Analysis | Explanatory variable | Coleoptera | Diptera | Hymenoptera | Hemiptera | Aranae | Other | Total number  of invertebrates |
| --- | --- | --- | --- | --- | --- | --- | --- | --- | --- |
| Pan trap | GLM | Humidity | χ^2^(1) = 1.11  p = 0.29 | χ^2^(1) = 1.56  p = 0.21 | χ^2^(1) = 0.019  p = 0.89 | χ^2^(1) = 2.83  p = 0. 093 | χ^2^(1) = 3.33  p = 0.068 | χ^2^(1) = 2.04  p = 0. 15 | χ^2^(1) = 0.77  p = 0. 38 |
|  | GLM | Population | **χ^2^(4) = 16.1**  **p = 0.003** | **χ^2^(4) = 15.54**  **p = 0.004** | **χ^2^(4) = 16.7**  **p = 0.002** | χ^2^(4) = 8.81  p = 0. 093 | χ^2^(4) = 3.35  p = 0.066 | χ^2^(4) = 5.28  p = 0. 26 | **χ^2^(4) = 13.32**  **p = 0. 01** |
|  | Regression | Herbivory intensity | R^2^ = -0.22, F_1,4_ = 0.281,  p = 0.63 | R^2^ = -0.14, F_1,4_ = 0.498,  p = 0.53 | R^2^ = 0.16, F_1,4_ = 1.77,  p = 0.28 | R^2^ = 0.17, F_1,4_ = 1.81,  p = 0.27 | R^2^ = -0.11, F_1,4_ = 0.613,  p = 0.49 | R^2^ = -0.08, F_1,4_ = 0.703,  p = 0.46 | R^2^ = -0.22,  F_1,4_ = 0.293,  p = 0.63 |
| Sticky trap | GLM | Humidity | **χ^2^(1) = 10.6**  **p = 0. 001** | χ**^2^(1) = 6.75**  **p = 0. 009** | **χ^2^(1) = 3.95**  **p = 0. 047** | **χ^2^ (1) = 925**  **p < 0.001** | NA | χ^2^(1) = 2.85  p = 0. 092 | **(χ^2^(1) = 12.9**  **p < 0.001** |
|  | Regression | Herbivory intensity | **R^2^ = 0.77,**  **F_1,4_ = 14.5,**  **p = 0.033** | R^2^ = 0.66,  F_1,4_ = 8.72,  p = 0.056 | R^2^ = 0.35,  F_1,4_ = 3.19,  p = 0.17 | R^2^ = 0.69,  F_1,4_ = 9.88,  p = 0.052 | NA | R^2^ = 0.61,  F_1,4_ = 7.12,  p = 0.076 | **R^2^ = 0.77,**  **F_1,4_ = 14.1,**  **p = 0.03** |

**Table S6.** Overview of results from perMANOVAs performed to compare beta diversity (Bray Curtis distances) in the bacterial and fungal communities (including data from

AMF-specific primers analyzed separately) in relation to four factors (Wet/dry habitat, Habitat, Population, Plant species). Habitat, population and wet/dry were compared between root samples of *S. dulcamara* whereas plant species was compared across all root samples. All analyses were run with 999 permutations.

| Factor | Microbial community | Diversity  metric | df | Pseudo-F | p (perm) |
| --- | --- | --- | --- | --- | --- |
| Wet vs. dry | Bacteria | Bray Curtis | 1 | 9.02 | 0.001 |
|  | Fungi | Bray Curtis | 1 | 8.21 | 0.001 |
|  | AMF | Bray Curtis | 1 | 5.71 | 0.001 |
| Habitat | Bacteria | Bray Curtis | 3 | 8.20 | 0.001 |
|  | Fungi | Bray Curtis | 3 | 5.58 | 0.001 |
|  | AMF | Bray Curtis | 3 | 4.28 | 0.001 |
| Population | Bacteria | Bray Curtis | 7 | 6.98 | 0.001 |
|  | Fungi | Bray Curtis | 7 | 4.30 | 0.001 |
|  | AMF | Bray Curtis | 7 | 4.21 | 0.001 |
| Plant species | Bacteria | Bray Curtis | 4 | 3.57 | 0.001 |
|  | Fungi | Bray Curtis | 4 | 2.39 | 0.001 |
|  | AMF | Bray Curtis | 4 | 3.68 | 0.001 |

**Table S7.** Pairwise comparisons from perMANOVAs performed to compare beta diversity (Bray Curtis distances) in the bacterial and fungal communities (including data from AMF-specific primers analyzed separately) in relation to the factors Habitat, Population and Plant species. Habitat and population were compared between root samples of *S. dulcamara* whereas plant species was compared across all root samples. All analyses were run with 999 permutations.

|  | Bacteria | | Fungi | | AMF | |
| --- | --- | --- | --- | --- | --- | --- |
|  | Pseudo-F | p adjusted | Pseudo-F | p adjusted | Pseudo-F | p adjusted |
| **Habitats** |  |  |  |  |  |  |
| Forest vs. urban | 7.14 | 0.001 | 6.11 | 0.001 | 3.85 | 0.002 |
| Forest vs. rural | 4.95 | 0.001 | 3.00 | 0.001 | 3.64 | 0.002 |
| Forest vs. beach | 10.5 | 0.001 | 7.26 | 0.001 | 6.21 | 0.002 |
| Urban vs. rural | 9.12 | 0.001 | 6.91 | 0.001 | 2.89 | 0.006 |
| Urban vs. beach | 9.63 | 0.001 | 4.95 | 0.001 | 3.15 | 0.002 |
| Rural vs. beach | 8.01 | 0.001 | 5.26 | 0.001 | 5.76 | 0.002 |
| **Populations** |  |  |  |  |  |  |
| Alnarp pond (F1) vs. Habo Ljung (F2) | 5.79 | 0.001 | 2.97 | 0.002 | 2.42 | 0.019 |
| Alnarp pond (F1) vs. Lund (U1) | 6.80 | 0.001 | 4.67 | 0.002 | 4.01 | 0.002 |
| Alnarp pond (F1) vs. Borgeby (R1) | 4.74 | 0.001 | 2.63 | 0.002 | 3.24 | 0.005 |
| Alnarp pond (F1) vs. Kämpinge (B2) | 8.52 | 0.001 | 5.30 | 0.002 | 5.50 | 0.002 |
| Alnarp pond (F1) vs. Lomma beach (B1) | 8.84 | 0.001 | 5.03 | 0.002 | 6.81 | 0.002 |
| Alnarp pond (F1) vs. Tvedöra (R2) | 8.39 | 0.001 | 3.87 | 0.002 | 3.43 | 0.006 |
| Alnarp pond (F1) vs. Malmö (U2) | 5.50 | 0.001 | 4.34 | 0.002 | 7.08 | 0.002 |
| Habo Ljung (F2) vs. Lund (U1) | 7.35 | 0.001 | 4.62 | 0.002 | 2.33 | 0.017 |
| Habo Ljung (F2) vs. Borgeby (R1) | 6.86 | 0.001 | 2.68 | 0.004 | 2.41 | 0.004 |
| Habo Ljung (F2) vs. Kämpinge (B2) | 6.90 | 0.001 | 5.81 | 0.002 | 3.00 | 0.003 |
| Habo Ljung (F2) vs. Lomma beach (B1) | 7.69 | 0.001 | 5.72 | 0.002 | 3.98 | 0.002 |
| Habo Ljung (F2) vs. Tvedöra (R2) | 6.85 | 0.001 | 2.98 | 0.002 | 4.49 | 0.002 |
| Habo Ljung (F2) vs. Malmö (U2) | 6.11 | 0.001 | 6.08 | 0.002 | 5.80 | 0.002 |
| Lund (U1) vs. Borgeby (R1) | 8.39 | 0.001 | 4.27 | 0.002 | 1.93 | 0.038 |
| Lund (U1) vs. Kämpinge (B2) | 7.10 | 0.001 | 3.91 | 0.002 | 2.97 | 0.002 |
| Lund (U1) vs. Lomma beach (B1) | 8.65 | 0.001 | 4.13 | 0.002 | 4.70 | 0.002 |
| Lund (U1) vs. Tvedöra (R2) | 11.4 | 0.001 | 5.44 | 0.002 | 3.86 | 0.009 |
| Lund (U1) vs. Malmö (U2) | 3.41 | 0.001 | 3.18 | 0.002 | 6.79 | 0.002 |
| Borgeby (R1) vs. Kämpinge (B2) | 7.18 | 0.001 | 4.24 | 0.002 | 2.56 | 0.005 |
| Borgeby (R1) vs. Lomma beach (B1) | 6.98 | 0.001 | 4.03 | 0.002 | 3.92 | 0.002 |
| Borgeby (R1) vs. Tvedöra (R2) | 8.10 | 0.001 | 2.70 | 0.003 | 3.10 | 0.007 |
| Borgeby (R1) vs. Malmö (U2) | 6.49 | 0.001 | 5.07 | 0.002 | 5.09 | 0.002 |
| Kämpinge (B2) vs. Lomma beach (B1) | 2.85 | 0.001 | 3.05 | 0.002 | 3.25 | 0.002 |
| Kämpinge (B2) vs. Tvedöra (R2) | 7.66 | 0.001 | 4.60 | 0.002 | 5.17 | 0.002 |
| Kämpinge (B2) vs. Malmö (U2) | 5.50 | 0.001 | 4.68 | 0.002 | 5.00 | 0.002 |
| Lomma beach (B1) vs. Tvedöra (R2) | 7.82 | 0.001 | 4.27 | 0.002 | 7.95 | 0.002 |
| Lomma beach (B1) vs. Malmö (U2) | 5.83 | 0.001 | 4.44 | 0.002 | 5.17 | 0.002 |
| Tvedöra (R2) vs. Malmö (U2) | 8.08 | 0.001 | 6.36 | 0.002 | 7.87 | 0.002 |
| **Plant species** |  |  |  |  |  |  |
| S. dulcamara vs. Lyc. europaeaus | 4.31 | 0.002 | 2.20 | 0.003 | 4.41 | 0.003 |
| S. dulcamara vs. P. lanceolata | 2.09 | 0.003 | 2.15 | 0.007 | 2.06 | 0.010 |
| S. dulcamara vs. Lys. vulgaris | 4.49 | 0.002 | 2.50 | 0.006 | 3.42 | 0.003 |
| S. dulcamara vs. A. vulgaris | 3.78 | 0.002 | 3.13 | 0.003 | 6.89 | 0.003 |
| Lyc. europaeaus vs. P. lanceolata | 2.87 | 0.028 | 2.21 | 0.037 | 1.32 | 0.10 |
| Lyc. europaeaus vs. Lys. vulgaris | 2.98 | 0.002 | 1.92 | 0.003 | 1.59 | 0.006 |
| Lyc. europaeaus vs. A. vulgaris | 4.45 | 0.002 | 2.26 | 0.003 | 2.24 | 0.003 |
| P. lanceolata vs. Lys. vulgaris | 1.96 | 0.076 | 1.98 | 0.030 | 1.13 | 0.19 |
| P. lanceolata vs. A. vulgaris | 1.79 | 0.025 | 1.96 | 0.013 | 1.34 | 0.093 |
| Lys. vulgaris vs. A. vulgaris | 3.17 | 0.002 | 1.40 | 0.044 | 1.68 | 0.014 |

**Table S8.** Pearson correlations between selected environmental and plant performance trait variables and the Bray Curtis distances for bacterial, fungal and AMF communities, respectively, tested with Mantel tests. Soil pH, plant dry matter and nutrients were analysed per *Solanum dulcamara* population (n = 8) and plant performance traits measured in the plants sampled for root microbiome analysis in August (n = 94 from eight populations). Significant differences ( p < 0.05) are shown in bold.

|  | Bacteria | | Fungi | | AMF | |
| --- | --- | --- | --- | --- | --- | --- |
| **Variable** | Correlation coefficient | p adjusted | Correlation coefficient | p adjusted | Correlation coefficient | p adjusted |
| Soil pH | **0.128** | **0.004** | **0.163** | **0.002** | -0.014 | 0.84 |
| Plant dry matter (%) | 0.073 | 0.13 | **0.157** | **0.002** | 0.094 | 0.11 |
| Plant N (g/kg) | **0.108** | **0.016** | **0.210** | **0.002** | 0.106 | 0.056 |
| Plant P (g/kg) | **0.141** | **0.014** | **0.249** | **0.002** | 0.084 | 0.11 |
| Plant K (g/kg) | **0.406** | **0.004** | **0.247** | **0.002** | **0.113** | **0.042** |
| Plant Ca (g/kg) | **0.278** | **0.004** | **0.196** | **0.002** | 0.082 | 0.078 |
| Plant Mg (g/kg) | **0.466** | **0.004** | **0.238** | **0.002** | **0.199** | **0.001** |
| Combined length all shoots (cm) | -0.053 | 0.91 | 0.015 | 0.40 | -0.104 | 0.96 |
| Dry weight above ground biomass (g) | -0.120 | 0.99 | 0.019 | 0.40 | -0.055 | 0.88 |
| Longest root (cm) | 0.041 | 0.22 | 0.062 | 0.078 | -0.028 | 0.88 |
| Number of flowers | 0.009 | 0.47 | 0.004 | 0.50 | -0.057 | 0.88 |
| Number of berries | 0.067 | 0.21 | 0.084 | 0.078 | 0.120 | 0.073 |
| Runner vs. whole plant (per plant id) | **0.096** | **0.042** | 0.067 | 0.056 | 0.086 | 0.16 |
| Herbivory exposure (per plant id) | 0.081 | 0.13 | -0.019 | 0.64 | 0.031 | 0.33 |

**Figure S1.** Box plots from leaf analysis identifying the percentage of leaf surface areas missing (= eaten by herbivores) in (a) eight *S. dulcamara* populations and b) across four habitats of these populations. c) Five leaves were collected from five plants in each population and analysed using image analysis.

**Figure S2.** Number of individual arthropods collected in five *S. dulcamara* populations with (a) sticky traps and (b) pan traps. Arthropods were classified to the taxonomic level order. The category Other combines specimens from various orders that were too few to statistically analyse on their own. Pan traps n: Alnarp pond (8), Borgeby (9), Lund (8), Habo Ljung (10), Kämpinge (9).

**Figure S3.** PCA Biplot visualising the association between soil and top shoot properties for eight *Solanum dulcamara* populations across four habitats.

**b**

**a**

**Figure S4.** Box plots of alpha diversity (Chao 1) of (a) bacterial and (b) total fungal communities in *S. dulcamara* roots from eight populations and four types of habitats. Different letters indicate significant (p < 0.05) differences. Wet populations: A = Alnarp pond (F1), B = Habo Ljung (F2) , D = Borgeby (R1), G = Tvedöra (R2); Dry populations: C = Lund (U1), E = Kämpinge (B2), F = Lomma beach (B1), H = Malmö (U2)
